# Effect of filling degree on particle segregation in a rotating drum - an experimental and numerical study

**DOI:** 10.1038/s41598-026-65443-2

**Published:** 2026-08-04

**Authors:** Theodoros Nestor Papapetrou, Martina Bieberle, Frank Barthel, Uwe Hampel, Gregory Lecrivain

**Affiliations:** 1 Institute of Fluid Dynamics, Helmholtz-Zentrum Dresden – Rossendorf, Bautzner Landstraße 400, 01328 Dresden, Germany; 2 Institute of Power Engineering, Chair of Imaging Techniques in Energy and Process Engineering, Technische Universität Dresden, 01069 Dresden, Germany

**Keywords:** Granular mixing in a rotating drum, Ultrafast X-ray computed tomography, Discrete element method, Camera, Filling degree effect, Engineering, Materials science, Mathematics and computing, Physics

## Abstract

Granular mixing, ubiquitous in industry, is difficult to observe. Ultrafast X-ray computed tomography is a relatively new technique that can be used to study rapid granular flows in opaque systems. A comparative study is here presented, where ultrafast X-ray computed tomography, camera and simulation with the Discrete Element Method are simultaneously used to investigate the radial segregation of 4-mm spherical polypropylene and glass beads in a rotating drum. The drum filling is varied from 20 to 50%. The granular flow and the associated segregation dynamics at the front-end wall and in the bulk are compared and good agreement among the three methods is found. Similar to unary systems, a transition from the rolling to the sliding regime, characterized by a critical dynamic angle of repose, is also reported.

## Introduction

Mixing of granular solids is found in diverse industrial applications, such as pharmaceuticals, food and concrete processing^1–3^. While the aim is to produce a spatially homogeneous blend, differences in particle properties, such as size^4–7^, density^8–10^ or shape^11,12^, result in spontaneous particle segregation during the granular flow^13,14^. In the case of a binary granular system in a rotating drum, as is the case here, rapid segregation dynamics in the radial direction^15,16^ are followed by much slower axial segregation^17,18^, forming complex three-dimensional segregation patterns^19–23^. We focus here on radial segregation.

Several experimental and numerical methods have been used to investigate the granular flow in a rotating drum. Camera imaging is a fast and easy method to generate high quality images. It is well-suited for quasi-2D drum experiments^10,24–26^. It can also be used to observe the bed through all transparent surfaces of a 3D drum^27,28^. It provides qualitative information about the flow regime^29^, the radial^25,26^ and axial^27,28,30^ segregation patterns, as well as quantitative information about the segregation dynamics on the visible surfaces^30–32^. With a camera, it is generally difficult to determine the trajectories of individual particles over long periods of time, because the particles eventually move to the bulk. With particle image velocimetry (PIV) or particle tracking velocimetry (PTV), it is possible to determine velocity fields in a quasi-2D drum^10,33^. Other non-invasive experimental techniques have been used to investigate particle segregation in rotating drums^34^. Among them, there exists magnetic resonance imaging (MRI), which produces time-averaged cross-sectional images of the bulk. MRI has been used to investigate steady-state segregation patterns^35,36^ and flow fields^37,38^, yet it is not fast enough to provide transient data^39^. Radioactive particle tracking (RPT) and positron emission particle tracking (PEPT) are further common techniques in the field^40^. They rely on radiation from tracer particles to track their positions and velocities. Steady-state parameters such as the active layer depth and the axial dispersion coefficient can be calculated from the reconstructed trajectories and velocity fields^4,41–43^. Simulations, often validated against camera data^9,25,44^, have become a good alternative to costly experiments, especially to study the bulk^45,46^. Both Eulerian, where the solid phases are modeled as a continuum^7,47^, and Lagrangian simulations with the Discrete Element Method (DEM), where the motion of each individual particle is calculated^6,27,48^, are common approaches. Simulations provide additional insight into the segregation patterns^46,49^, mixing dynamics^50,51^, particle trajectories^9^ and velocity fields^52,53^. In addition, they are used to obtain information unavailable from experiments, such as particle forces^54–56^ and bulk stress^57^, or to study granular flow with on-top phenomena, such as heat transfer^58–61^.

Lately, granular segregation has been studied with X-ray computed tomography too. Gajjar et al.^12^ used time-lapse X-ray computed tomography to visualize the Brazil-nut effect in real time. In this line of research, a relatively new technique, namely the ultrafast X-ray computed tomography (UFXCT), is of increasing interest for the visualization of rapid granular flows. It has only been used a few times for granular systems. With a temporal resolution up to 8 kHz^62^, Stannarius et al.^63^ used it to study granular flow during silo discharge. In our recent work^64^, we showed that UFXCT is also well applicable to visualize the transient segregation patterns in the bulk of a rotating drum. Yet, there exist no studies where UFXCT data are compared to other bulk data from experiments, simulations or theories. In this context, we use UFXCT, camera and DEM to investigate the effect of the filling degree on both the granular flow and the segregation dynamics in a rotating drum. In a system driven by density difference, a central core filled with heavy particles forms. For a drum which is more than half filled, the granular core remains unmixed^65^. When the drum is less than half filled, which is here of particular interest, the segregation core becomes more clearly defined with increasing filling^48,50^. This trend was observed both in simulations and at the circular cap of a drum^30^. Also, Khakhar et al.^25^ reported segregation cores of the same relative size in a half filled and a quarter filled quasi-2D drum.

The link between the filling degree and flow velocity field has also been studied. In the rolling and cascading flow regimes, commonly found in industry, the particle bed is divided into a lower passive layer, where the particles move with the drum, and an upper active layer^29,45^. In the active layer, mixing and segregation occur. This in turn leads to the formation of the segregation core, whose shape and size is function of, among others, particle properties and operating parameters^26,66^. In unary systems, camera imaging and Eulerian simulations have shown that, for filling degrees in the range of 15–30%, an increase in the filling degree results in a thicker active layer^53,67^. Chou et al.^68^ studied a broader range of filling degrees (14–50%) and observed the opposite trend. A higher filling led to a higher dynamic angle of repose and a thinner active layer. They also reported wall slip at low filling because of reduced particle-wall friction. Parker et al.^41^ also observed slipping at the wall for a filling degree of about 35% in their PEPT experiments. At even lower filling degrees, the flow can transition to the sliding regime, where the entire bed slides on the drum and mixing is minimal^69^. This regime is therefore undesirable for industry, so it is not as widely studied^70^. Mellmann^29^, however, studied the transition between rolling to sliding regime for unary systems and found that the sliding regime only occurs for sufficiently small drum speed and wall friction.

The objectives of this work are twofold. First, we seek to compare UFXCT data to DEM data and so, showcase the advantages and limits of UFXCT compared to other techniques. We also make use of a camera to validate the DEM data taken at the transparent front plane. Second, we investigate the effect of the filling degree on the velocity field and the flow regime. In the following, we present results on the transient and steady-state radial segregation patterns in two axial planes. We also determine granular velocity fields from images taken with the three methods. The velocity fields provide insight into the particle-wall interactions and the flow regime. This was not done in our previous work. Concentration fields are also used to further compare the three methods and to draw conclusions about how the filling degree correlates with the segregation patterns.

## Methods

### Experimental scenarios

**Fig. 1:**
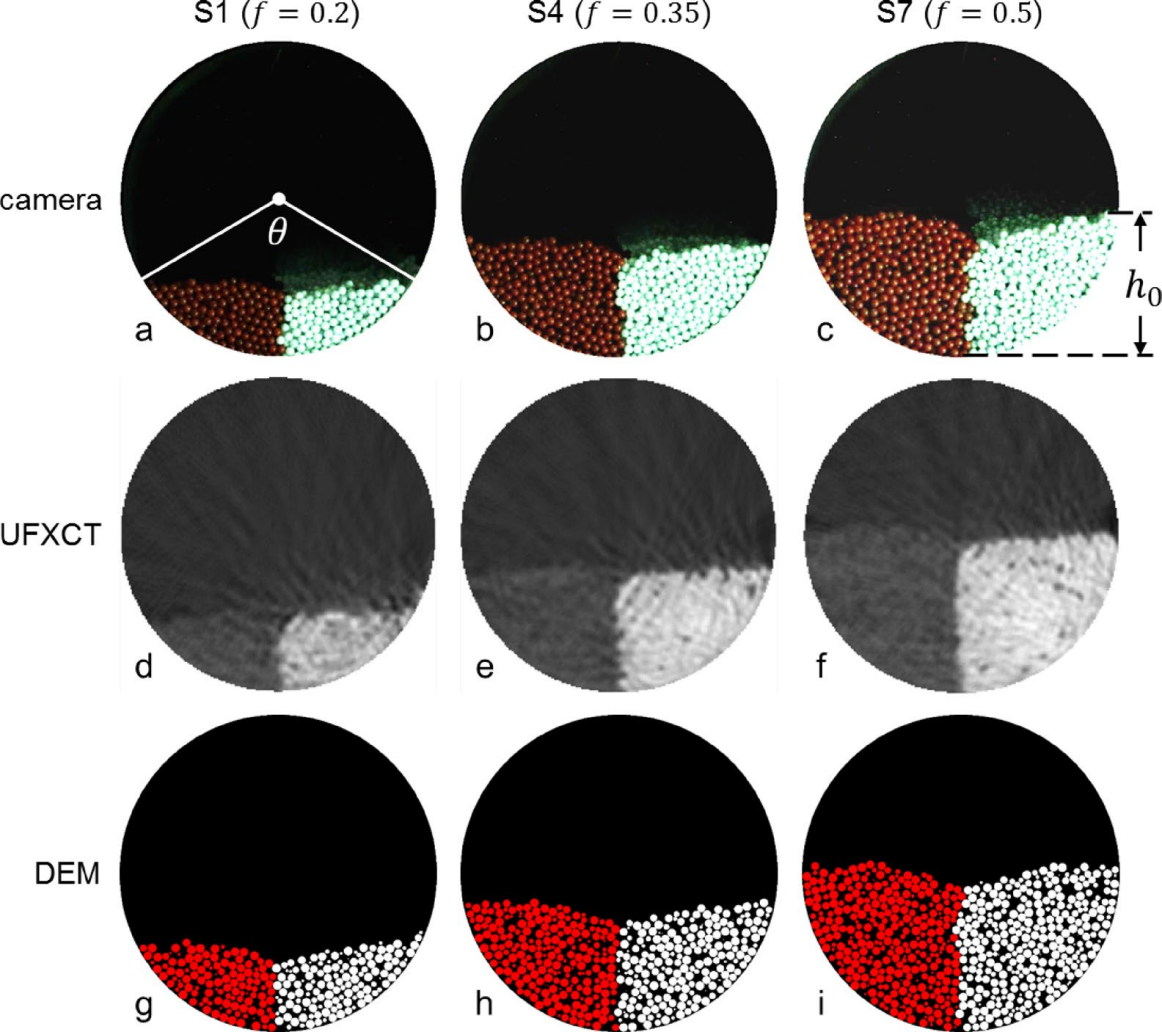
Images of the bed at *\:t=0* s for the three experimental scenarios S1, S4 and S7. Top row (a-c): camera images at the front-end (*\:z/L=0)*. Middle row (d-f): UFXCT images in the bulk (*\:z/L=0.5)*. Bottom row (g-h): DEM cuts through the bulk (*\:z/L=0.5*). Polypropylene particles are red and glass particles white. *\:f* is the filling degree and *\:L* the length of the drum.

The present experimental set-up consists of a horizontal rotating drum partly filled with a binary bed of spherical particles. A detailed description can be found in the authors’ previous works^64,71^. For the sake of conciseness, only the most salient features of the set-up are here presented. The particles in each granular species have the same diameter, denoted by the lower letter $\:{d}_{p}$ and $\:{d}_{g}$ and only differ in density and color. The polypropylene particles, hereafter associated with the subscript (*\:p*) are red and the glass particles with subscript (*\:g*) are white. Initially, that is at time *\:t=0* s, the bed is in a side-by-side segregated state as shown in Fig. 1. The two granular species occupy the same volume. The total volume of granular material in the drum divided by the inner drum volume defines the filling degree (*\:f*). In practice, *\:f* is calculated from the cross-sectional images by fitting a straight line to the free surface of the bed. The filling degree in Fig. 1 is calculated with the help of elementary trigonometry as

1 $$\:f=\frac{\theta\:-\mathrm{sin}\theta\:}{2\pi\:},$$

where $\:\theta\:=2{\mathrm{cos}}^{-1}(1-{h}_{0}/R)$ is the filling angle^24^, itself function of the initial maximal height $\:{h}_{0}$ of the granular bed and the inner radius *\:R* of the drum.

The drum rotates about its long axis with a constant rotational speed, denoted by $\:{n}_{0}$. In each experiment, the drum accelerates and decelerates for about 0.1 s. This time is minimal and corresponds to less than 5% of a full drum rotation. Typically, three to five rotations are needed to reach the steady-state (see Sect. 2.5 for the precise definition of the steady-state). The drum speed is converted to a non-dimensional Froude number given by

2 $$\:Fr=\frac{{\omega\:}_{0}^{2}R}{g},$$

where $\:{\omega\:}_{0}=2\pi\:{n}_{0}$ is the drum angular velocity and *\:g* the gravity. The filling degree and the Froude numbers give an indication about the flow regime^29^. Here we consider the rolling and cascading regimes, because they are most relevant for mixing and there is good availability of literature data^29^. In the rolling regime, the free surface of the bed is a flat, inclined plane. In the cascading regime, the free surface turns into a sigmoid. We also consider the sliding regime, which occurs for low filling degrees, as will be discussed in the results. Like in the rolling regime, the free surface in the sliding regime is flat. The relevant parameters related to the present experimental set-up are summarized in Table 1. We note that the two particle species differ slightly in their diameter. The glass and polypropylene spheres have a mean diameter of 4.00 mm and 3.97 mm, respectively. The resulting diameter ratio of 1.008 is close to unity and lies well outside the range typically considered in size-driven particle segregation studies^72^. The two species also differ in material and likely in surface roughness, which may influence the segregation dynamics^73^. Disentangling these effects would require a dedicated study and lies beyond the scope of this work. With a density ratio of 2.8, we expect the density difference to be the main driver behind the radial segregation dynamics presented here.

**Table 1:** Experimental parameters.

Parameter	Symbol	Value	Units
Drum length	*\:L*	300	mm
Drum inner diameter	*\:D*	144	mm
Rotational speed	$\:{n}_{0}$	27	rpm
Froude number	*\:Fr*	5.9∙10^− 2^	
Polypropylene particle diameter	$\:{d}_{p}$	3.97 ± 0.13	mm
Glass particle diameter	$\:{d}_{g}$	4.00 ± 0.30	mm
Polypropylene particle density	$\:{\rho\:}_{p}$	0.9	g/cm^3^
Glass particle density	$\:{\:\rho\:}_{g}$	2.5	g/cm^3^
Polypropylene mass error	$\:\delta\:{m}_{p}$	0.03	g
Glass mass error	$\:\delta\:{m}_{g}$	0.03	g
Polypropylene volume fraction in bed	$\:{v}_{p}$	0.5	
Glass volume fraction in bed	$\:{v}_{g}$	0.5	
Bed packing fraction	$\:{f}_{p}$	0.64	
Initial drum acceleration time		0.1	s
Drum deceleration time		0.1	s

Three scenarios associated with three different filling degrees, that are $\:f=\{0.2,\:0.35,\:0.5\}$, are studied by means of camera, UFXCT and DEM simulations. Additional scenarios supplemented with DEM are also presented. In each scenario, the numbers $\:{n}_{p}$ and $\:{n}_{g}$ of polypropylene and glass particles, used as input in the DEM simulations, are identical. They are estimated from the granular masses $\:{m}_{p}$ and $\:{m}_{g}$, the particle diameters and the material densities of polypropylene and glass particles in the drum (See Tables 1 and 2). Here, $\:{n}_{p}$ varies from about 9∙10^3^ in scenario S1 to about 24∙10^3^ in scenario S7.

**Table 2:** Investigated scenarios. $\:{m}_{p}$ and $\:{m}_{g}$ are the mass of polypropylene and glass particles in the drum. They are expressed in grams.

Scenario	Filling degree (*\:f*)	$\:{m}_{p}$/g	$\:{m}_{g}$/g	Methods
S1	0.20	281.42	781.73	Camera, UFXCT, DEM
S2	0.25	351.78	977.16	DEM
S3	0.30	422.13	1172.59	DEM
S4	0.35	492.49	1368.03	Camera, UFXCT, DEM
S5	0.40	562.84	1563.46	DEM
S6	0.45	633.20	1758.89	DEM
S7	0.50	703.56	1954.32	Camera, UFXCT, DEM

Irrespective of the method, the data are obtained over the course of 30 rotations. We distinguish between front-end and bulk data. The experimental front-end raw data are color images (Fig. 1a-c) taken with a camera through the transparent front cap of the drum. The experimental bulk data are gray cross-sectional images (Fig. 1d-f) taken with the Ultrafast X-ray Computed Tomography (UFXCT). A higher grey value indicates here a higher material density. In short, 25 front-end camera images are taken each second. Each camera image is transformed into a ternary one with white (glass), red (polypropylene) and black (air) pixels. The particle diameter is discretized into 16 ± 1 pixels. In the bulk, 1000 X-ray images are taken each second. Due to the random noise inherent in the UFXCT method^74^, the average of every ten images is taken, reducing the effective frequency to 100 Hz without much loss of information. The resulting UFXCT images are also transformed into ternary ones. The particle diameter is here discretized into 5 ± 1 pixels. More information on camera, UFXCT image processing and artefact correction is available in the authors’ previous works^64,71,75^.

### DEM simulations

The particle system is also studied with the discrete element method (DEM)^76^. The open source simulation software LIGGGHTS^77^ is used. The translational ($\:{\boldsymbol{V}}_{i}$) and angular ($\:{\boldsymbol{\varOmega\:}}_{i}$) velocities of each particle, hereafter denoted by the letter *\:i*, are calculated by solving Newton’s laws of motion^78–80^. $\:{\boldsymbol{V}}_{i}$ and $\:{\boldsymbol{\varOmega\:}}_{i}$ are given by

3 $$\:{m}_{i}\frac{\mathrm{d}{\boldsymbol{V}}_{i}}{\mathrm{d}t}={m}_{i}\boldsymbol{g}+\sum\:_{j=1}^{{N}_{i}}{\boldsymbol{F}}_{ij}$$

and

4 $$\:{\boldsymbol{I}}_{i}\frac{\mathrm{d}{\boldsymbol{\varOmega\:}}_{i}}{\mathrm{d}t}=\sum\:_{j=1}^{{N}_{i}}\left({\boldsymbol{R}}_{ij}\times\:{\boldsymbol{F}}_{ij}+{\boldsymbol{M}}_{ij}^{r}\right),$$

where $\:{m}_{i}$ is the mass of particle *\:i*, *\:t* the time, $\:\boldsymbol{g}$ the gravity vector, $\:{N}_{i}$ the number of particles in contact with *\:i*, $\:{\boldsymbol{F}}_{ij}$ the contact force exerted by particle *\:j* on *\:i*, $\:{\boldsymbol{I}}_{i}$ the moment of inertia of *\:i*, $\:{\boldsymbol{R}}_{ij}$ the vector pointing from the center of particle *\:i* to the point of contact with *\:j* and $\:{\boldsymbol{M}}_{ij}^{r}$ the rolling friction torque^81^. The position $\:{\boldsymbol{X}}_{i}={\left({X}_{i}\:{Y}_{i}\:{Z}_{i}\right)}^{\top\:}$ of particle *\:i* is given by

5 $$\:{\boldsymbol{V}}_{i}=\frac{\mathrm{d}{\boldsymbol{X}}_{i}}{\mathrm{d}t}.$$

The particle orientation can be calculated from the angular velocity. The relevant equations, which can be found e.g. in^82,83^, are more complicated that Eq. (5) and for the sake of conciseness, we do not explicitly write them. The contact force ($\:{\boldsymbol{F}}_{ij}$) in Eqs. (3) and (4) is the sum of a normal ($\:{\boldsymbol{F}}_{ij}^{n}$) and a tangential ($\:{\boldsymbol{F}}_{ij}^{t}$) component,

6 $$\:{\boldsymbol{F}}_{ij}={\boldsymbol{F}}_{ij}^{n}+{\boldsymbol{F}}_{ij}^{t}.$$

The two force components in Eq. (6) are calculated with the nonlinear Hertz-Mindlin model^84^, which is commonly used in the literature^5,27,85,86^. In this model, the normal force is decomposed into a spring and a damping force as

7 $$\:{\boldsymbol{F}}_{ij}^{n}={k}_{n}{\boldsymbol{\delta\:}}_{ij}^{n}-{\gamma\:}_{n}{\boldsymbol{v}}_{ij}^{n},$$

where $\:{k}_{n}=4{E}^{\mathrm{*}}{\left({R}^{\mathrm{*}}\parallel{\boldsymbol{\delta\:}}_{ij}^{n}\parallel\right)}^{0.5}/3$ is the normal elastic constant, $\:{\boldsymbol{\delta\:}}_{ij}^{n}$ the normal overlap between the particles, $\:{\gamma\:}_{n}=$ $\:-\beta\:{\left(5{k}_{n}{m}^{\mathrm{*}}\right)}^{0.5}$ the normal viscoelastic damping constant and $\:{\boldsymbol{v}}_{ij}^{n}$ the normal relative velocity. Here $\:{E}^{\mathrm{*}}={\left(\left(1-{\nu\:}_{i}^{2}\right){E}_{i}^{-1}+\left(1-{\nu\:}_{j}^{2}\right){E}_{j}^{-1}\right)}^{-1}$, $\:{R}^{\mathrm{*}}={\left({R}_{i}^{-1}+{R}_{j}^{-1}\right)}^{-1}$ and $\:{m}^{\mathrm{*}}={\left({m}_{i}^{-1}+{m}_{j}^{-1}\right)}^{-1}$ are the equivalent Young’s modulus, radius and mass respectively, and $\:{\nu\:}_{i}$, $\:{E}_{i}$ and $\:{R}_{i}$ the Poisson’s ratio, Young’s modulus and radius of particle *\:i*, respectively. For the drum surfaces, both radius and mass are considered infinite. $\:\beta\:$ is given by

8 $$\:\beta\:=\mathrm{ln}\left({e}_{ij}\right){\left({\mathrm{ln}}^{2}\left({e}_{ij}\right)+{\pi\:}^{2}\right)}^{-0.5},$$

where $\:{e}_{ij}$ is the restitution coefficient. The tangential force is similarly decomposed into a shear and a damping force as

9 $$\:{\boldsymbol{F}}_{ij}^{t}={k}_{t}{\boldsymbol{\delta\:}}_{ij}^{t}-{\gamma\:}_{t}{\boldsymbol{v}}_{ij}^{t},$$

where $\:{k}_{t}=8{G}^{\mathrm{*}}{\left({R}^{\mathrm{*}}\parallel{\boldsymbol{\delta\:}}_{ij}^{n}\parallel\right)}^{0.5}$ is the tangential elastic constant, $\:{\boldsymbol{\delta\:}}_{ij}^{t}=\underset{coll}{\overset{}{\int\:}}{\boldsymbol{v}}_{ij}^{t}\mathrm{d}t$ the tangential overlap between the particles^87^, $\:{\gamma\:}_{t}=-2\beta\:{\left(5{k}_{t}{m}^{\mathrm{*}}/6\right)}^{0.5}$ the tangential viscoelastic damping constant, $\:{\boldsymbol{v}}_{ij}^{t}$ the tangential relative velocity and $\:{G}^{\mathrm{*}}={\left(2\left(2-{\nu\:}_{i}\right)\left(1+{\nu\:}_{i}\right){E}_{i}^{-1}+2\left(2-{\nu\:}_{j}\right)\left(1+{\nu\:}_{j}\right){E}_{j}^{-1}\right)}^{-1}$ the equivalent shear modulus. Resistance to slipping is modeled by truncating the tangential force according to Coulomb’s law of friction, so that $\:\parallel{\boldsymbol{F}}_{ij}^{t}\parallel\le\:{\mu\:}_{ij}\parallel{\boldsymbol{F}}_{ij}^{n}\parallel$, where $\:{\mu\:}_{ij}$ is the friction coefficient. A rolling friction torque ($\:{\boldsymbol{M}}_{ij}^{r}$) in Eq. (4) is added to represent the energy loss during rotation. A version of the elastic-plastic spring-dashpot model described by Ai et al.^88^ is used for the frictional torque, namely EPSD2 in LIGGGHTS. In this model, the rolling stiffness ($\:{k}_{r}$) is calculated with the relation suggested by Iwashita et al.^89^, $\:{k}_{r}={k}_{t}{{R}^{\mathrm{*}}}^{2}$, and the viscous damping torque described by Ai et al.^88^ is disabled. The only parameter in this model is the rolling friction coefficient ($\:{\mu\:}_{ij}^{r}$), used to truncate the frictional torque in a similar way to the tangential force, that is $\:\parallel{\boldsymbol{M}}_{ij}^{r}\parallel\le\:{\mu\:}_{ij}^{r}{R}^{\mathrm{*}}\parallel{\boldsymbol{F}}_{ij}^{n}\parallel$.

Table 3 shows the DEM parameters used in the presented simulations. All walls of the drum are taken to be of the same material, that is PMMA. The material properties of the PMMA drum, that are the Young’s modulus and Poisson’s ratio, are taken from literature^90^. The material properties of glass and polypropylene are taken from the manufacturers’ product specifications. The Young’s moduli for all three materials in the simulation were set to values hundred times smaller than their real counterparts. This is commonly done to artificially increase the collision durations and allow a greater discretization time step^52,91^. This in turn reduces the computational time with little, if any, adverse effect on the granular flow properties^92,93^. The restitution, friction and rolling friction coefficients are identical for all material pairs and estimated from typical ranges found in the literature^52,86,94^. We believe this is sufficient, as the DEM simulation is mainly used to provide bulk data for direct comparison with UFXCT. For more accurate results, a calibration method^93,95,96^ could have been used to estimate these coefficients. However, this would require considerable time and effort, especially for multiple flow regimes and transitions between regimes, without changing the message of the study significantly.

To initialize the particle bed, a virtual divider has been used. Prior to removing the divider, the drum slowly rotates a few times and so, a naturally and randomly packed bed illustrated in Fig. 1g-i is achieved. At time *\:t=0* s, the particle bed in the DEM is also in a side-by-side segregated state and its free surface is horizontal. We process the DEM data at a frequency matching the effective frequency of the UFXCT, that is 100 Hz. In each cross-sectional DEM image, the particle diameter is discretized into 30 pixels.

**Table 3:** DEM parameters.

Parameter	Symbol	Value	Units
Time step	$\:{\Delta\:}t$	4∙10^− 6^	s
Polypropylene particle Young’s modulus	$\:{E}_{p}$	1.3∙10^7^	Pa
Glass particle Young’s modulus	$\:{E}_{g}$	6.5∙10^8^	Pa
Drum wall Young’s modulus	$\:{E}_{w}$	2.45∙10^7^	Pa
Polypropylene particle Poisson’s ratio	$\:{\nu\:}_{p}$	0.42	
Glass particle Poisson’s ratio	$\:{\nu\:}_{g}$	0.22	
Drum wall Poisson’s ratio	$\:{\nu\:}_{w}$	0.36	
Restitution coefficient	$\:{e}_{ij}$	0.8	
Friction coefficient	$\:{\mu\:}_{ij}$	0.4	
Rolling friction coefficient	$\:{\mu\:}_{ij}^{r}$	5∙10^− 3^	

### Coordinate system

**Fig. 2:**
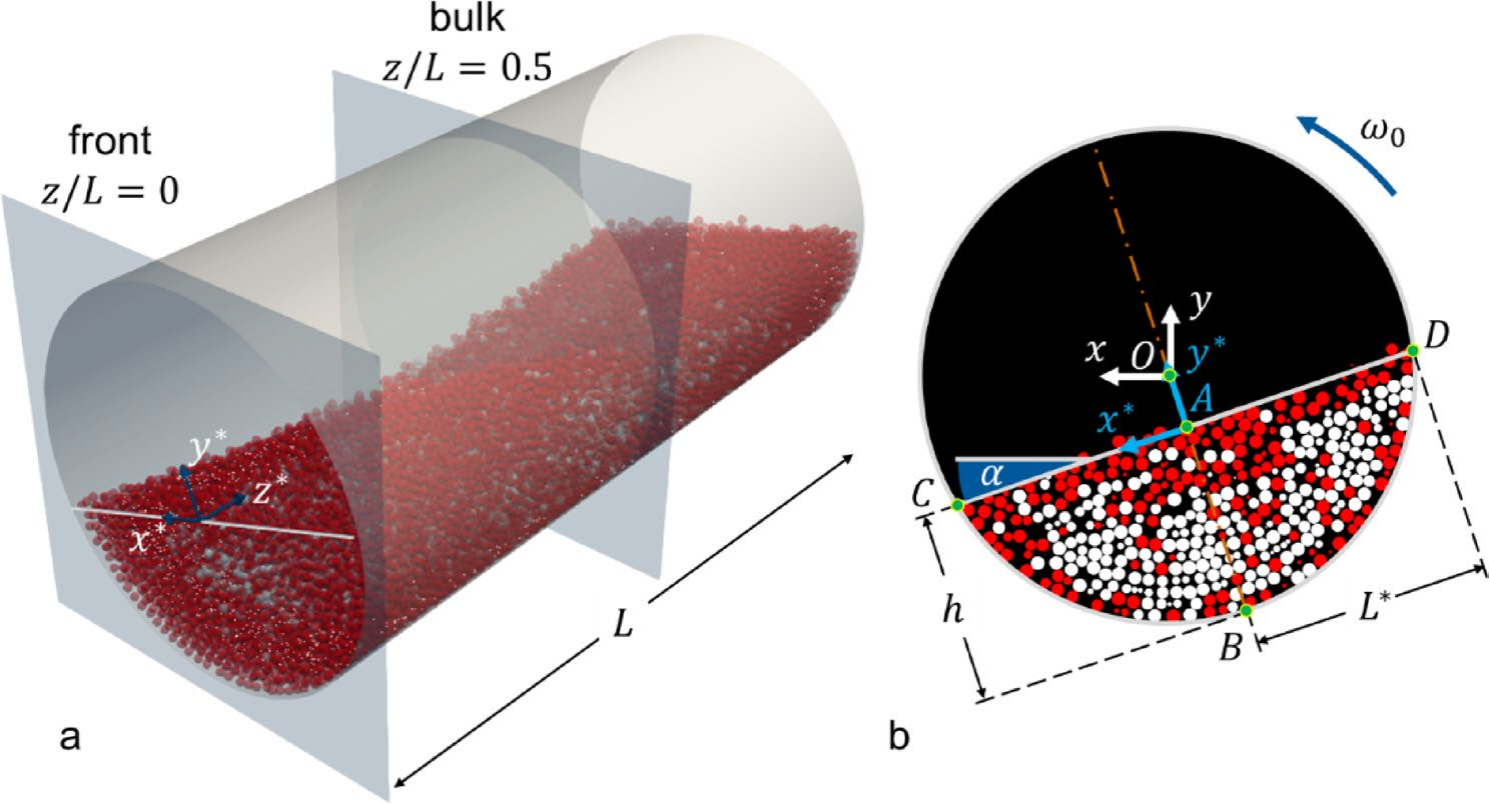
Three-dimensional representation of the drum and the particle bed in the steady-state. The two *\:z*-planes of interest (front, bulk) are highlighted. The drum frame is shown at the front-end plane **(a)**. Two-dimensional representation of the two coordinate systems at axial depth *\:z* **(b)**.

Two orthogonal coordinate systems, namely the inertial frame $\:\left(O,\widehat{\boldsymbol{x}},\widehat{\boldsymbol{y}},\widehat{\boldsymbol{z}}\right)$ and the drum frame $\:\left(A,{\widehat{\boldsymbol{x}}}^{\boldsymbol{*}},{\widehat{\boldsymbol{y}}}^{\boldsymbol{*}},{\widehat{\boldsymbol{z}}}^{\boldsymbol{*}}\right)$, are used to process and plot the data. The unit vectors $\:\widehat{\boldsymbol{z}}$ and $\:{\widehat{\boldsymbol{z}}}^{\boldsymbol{*}}$ are equal and align with the drum long axis. They point from the front-end (*\:z/L=0*) to the bulk (*\:z/L=0.5*). In the inertial frame, the *\:z* axis coincides with the drum long axis. The origin, *\:O*, is the intersection point of the drum long axis with the front-end plane. The unit vector $\:\widehat{\boldsymbol{y}}$ points upwards opposite to gravity and $\:\widehat{\boldsymbol{x}}=\widehat{\boldsymbol{y}}\times\:\widehat{\boldsymbol{z}}$. The drum frame, shown in Fig. 2a, is more convenient for data related to velocity fields and is defined for each axial depth (*\:z*). For a given axial depth, the straight line segment (*\:CD*) shown in Fig. 2b is fitted to the granular free surface. This is also done in the cascading regime, where the free surface is a sigmoid. The midpoint of *\:CD*, denoted by *\:A*, is the origin of the drum frame. The direction of the flow defines the orientation of the unit vector $\:{\widehat{\boldsymbol{x}}}^{\boldsymbol{*}}=AC/\left|AC\right|$. The unit vector $\:{\widehat{\boldsymbol{y}}}^{\boldsymbol{*}}={\widehat{\boldsymbol{z}}}^{\boldsymbol{*}}\times\:{\widehat{\boldsymbol{x}}}^{\boldsymbol{*}}$ points towards the air. The line segment *\:AB*, crossing the particle bed along $\:{\widehat{\boldsymbol{y}}}^{\boldsymbol{*}}$, connects the free surface to the drum wall at point *\:B*. We define the height of the particle bed $\:h=\left|AB\right|$, the free surface half-length $\:{L}^{*}=\left|AD\right|=\sqrt{h\left(D-h\right)}$, and the dynamic angle of repose $\:\alpha\:$ between the unit vectors $\:\widehat{\boldsymbol{x}}$ and $\:{\widehat{\boldsymbol{x}}}^{\boldsymbol{*}}$. The two coordinate systems are related to each other by

10 $$\:{\boldsymbol{x}}^{\boldsymbol{*}}=\left(\begin{array}{ccc}\mathrm{cos}\left(\alpha\:\right)&\:-\mathrm{sin}\left(\alpha\:\right)&\:0\\\:\mathrm{sin}\left(\alpha\:\right)&\:\mathrm{cos}\left(\alpha\:\right)&\:0\\\:0&\:0&\:1\end{array}\right)\boldsymbol{x}+\left(\begin{array}{c}0\\\:R-h\\\:0\end{array}\right),$$

where $\:\boldsymbol{x}={\left(x\:y\:z\right)}^{\top\:}$ and $\:{\boldsymbol{x}}^{\boldsymbol{*}}={\left({x}^{*}\:{y}^{*}\:{z}^{*}\right)}^{\top\:}$ are the space coordinates of the inertial and the drum frame respectively.

### Data processing

Irrespective of the acquisition method, be it camera, UFXCT, or DEM, each image pixel is associated with only one of the three phases, namely air, polypropylene or glass. It is useful to define three Eulerian fields $\:{c}_{a}\left(\boldsymbol{x},t\right)$, $\:{c}_{p}\left(\boldsymbol{x},t\right)$ and $\:{c}_{g}\left(\boldsymbol{x},t\right)$ associated with each phase in the drum. Each phase field equals 1 if the corresponding phase is found at the pixel position and 0 otherwise. We can write

11 $$\:{c}_{p}+{c}_{g}+{c}_{a}=1.$$

The local concentration of polypropylene relative to the granular mass at position $\:\boldsymbol{x}$ and time *\:t* is given by

12 $$\:c\left(\boldsymbol{x},t\right)=\frac{{c}_{p}}{{c}_{p}+{c}_{g}}.$$

Because $\:{c}_{a}\mathrm{\:=\:1}$ in pure air, *\:c* can be left undefined when the denominator in Eq. (12) equals zero. All concentration information is therefore contained in the two fields $\:{c}_{a}$ and *\:c*, which both take the values zero or unity only.

In the following analysis, the instantaneous state of the granular bed is characterized in each cross-sectional image by two integral values, that are the spatially averaged polypropylene concentration $\:\stackrel{-}{c}\left(z,t\right)$ and the mixing index $\:M\left(z,t\right)$. Both $\:\stackrel{-}{c}$ and *\:M* are dimensionless integral parameters, that are averaged in each *\:z*-plane, that is each plane normal to the drum long axis (see Fig. 2). Accordingly, the spatially averaged polypropylene concentration $\:\stackrel{-}{c}$ is calculated for each cross-sectional image as

13 $$\:\stackrel{-}{c}\left({z}_{0},t\right)=\frac{\underset{\mathbb{D}\left({z}_{0}\right)}{\overset{}{\iint\:}}{c}_{p}\mathrm{d}x\mathrm{d}y}{\underset{\mathbb{D}\left({z}_{0}\right)}{\overset{}{\iint\:}}\left({c}_{p}+{c}_{g}\right)\mathrm{d}x\mathrm{d}y}\approx\:\frac{{N}_{p}}{{N}_{p}+{N}_{g}},$$

where $\:z={z}_{0}$ is the drum depth, $\:\mathbb{D}\left({z}_{0}\right)=\left\{\boldsymbol{x}|{x}^{2}+{y}^{2}\le\:{R}^{2},\:z={z}_{0}\right\}$ the drum cross-section at depth $\:{z}_{0}$ and $\:{N}_{p}$ and $\:{N}_{g}$ are the total number of polypropylene (red) and glass (white) pixels in the image taken at time *\:t*. With this definition, $\:\stackrel{-}{c}$ reflects the ratio of polypropylene particles to the total number of particles in each image.

The mixing index *\:M* also ranges from zero to unity. *\:M=0* indicates complete segregation and *\:M=1* a homogeneous mixture. Different formulations can be found in the literature^2,50,97–99^. The one introduced in^71^, which extends on the contact-based method originally suggested by van Pyuvelde et al.^98^, is presently used. The method can be readily applied to camera, UFXCT or DEM images. The mixing index is calculated for each cross-sectional image as

14 $$\:M(z,t)=\frac{{L}_{c}-{L}_{\mathrm{m}\mathrm{i}\mathrm{n}}}{{L}_{\mathrm{m}\mathrm{a}\mathrm{x}}-{L}_{\mathrm{m}\mathrm{i}\mathrm{n}}},$$

where $\:{L}_{c}$ is the total length of all red/white contact lines in the image and $\:{L}_{\mathrm{m}\mathrm{i}\mathrm{n}}$ and $\:{L}_{\mathrm{m}\mathrm{a}\mathrm{x}}$ the minimum and maximum contact lengths normalizing *\:M*. All three lengths in Eq. (14) are calculated from each image. $\:{L}_{c}$ is calculated directly from the number of contacts between red and white pixels in the image, whereas $\:{L}_{\mathrm{m}\mathrm{i}\mathrm{n}}$ and $\:{L}_{\mathrm{m}\mathrm{a}\mathrm{x}}$ are functions of the particle diameter ($\:{d}_{p}$, $\:{d}_{g}$), the bed height (*\:h*) and the polypropylene concentration ($\:\stackrel{-}{c}$) in the image. Further details on the above procedure along with its validation are found in^71^. Because the two integral values $\:\stackrel{-}{c}$ and *\:M* in different bulk planes are close to one another, only those in the vertical mid-plane, that is *\:z/L=0.5*, are shown. We note that UFXCT measurement at the front-end is not possible because the PMMA circular cap of the drum interferes too much in terms of attenuation.

### Time-averaging

Hereafter, the infinity symbol is introduced to denote the time averaging in the steady-state, that is here between the times $\:{t}_{0}=10/{n}_{0}$, that is the 10th drum rotation, and $\:{t}_{1}=30/{n}_{0}$. As will be shown in the results, the polypropylene concentration $\:\stackrel{-}{c}$ reaches a steady-state by $\:t={t}_{0}$ in all scenarios. In the steady-state, $\:\stackrel{-}{c}$ is characterized by a mean value $\:{\stackrel{-}{c}}_{\infty\:}$ superimposed with a fluctuating component, that is reminiscent of turbulence in fluids^64^. The time-averaging of the quantity *\:Q* performed in the steady-state is given by

15 $$\:{Q}_{\infty\:}\left(\boldsymbol{x}\right)=\frac{1}{{t}_{1}-{t}_{0}}\underset{{t}_{0}}{\overset{{t}_{1}}{\int\:}}Q(\boldsymbol{x},t)\mathrm{d}t\approx\:\frac{1}{{N}_{S}}\sum\:_{{t}_{i}={t}_{0}}^{{t}_{1}}Q\left(\boldsymbol{x},{t}_{i}\right),$$

where $\:{N}_{S}$ is the number of images in the time interval $\:{t}_{0}$ to $\:{t}_{1}$. In practice, the number of images used for the time averaging in the order of $\:{N}_{S}\approx\:1000$ for camera and *\:5000* for the UFXCT and DEM data. A greater number of images does not change the results.

#### Concentration field

We introduce $\:{c}_{a\infty\:}$, $\:{c}_{p\infty\:}$ and $\:{c}_{g\infty\:}$ to represent the time-averaging of the phase fields related to air, polypropylene and glass. Accordingly, the steady-state polypropylene concentration field $\:{c}_{\infty\:}$ is given by

16 $$\:{c}_{\infty\:}\left(\boldsymbol{x}\right)=\frac{{c}_{p\infty\:}}{{c}_{p\infty\:}+{c}_{g\infty\:}}.$$

**Fig. 3:**
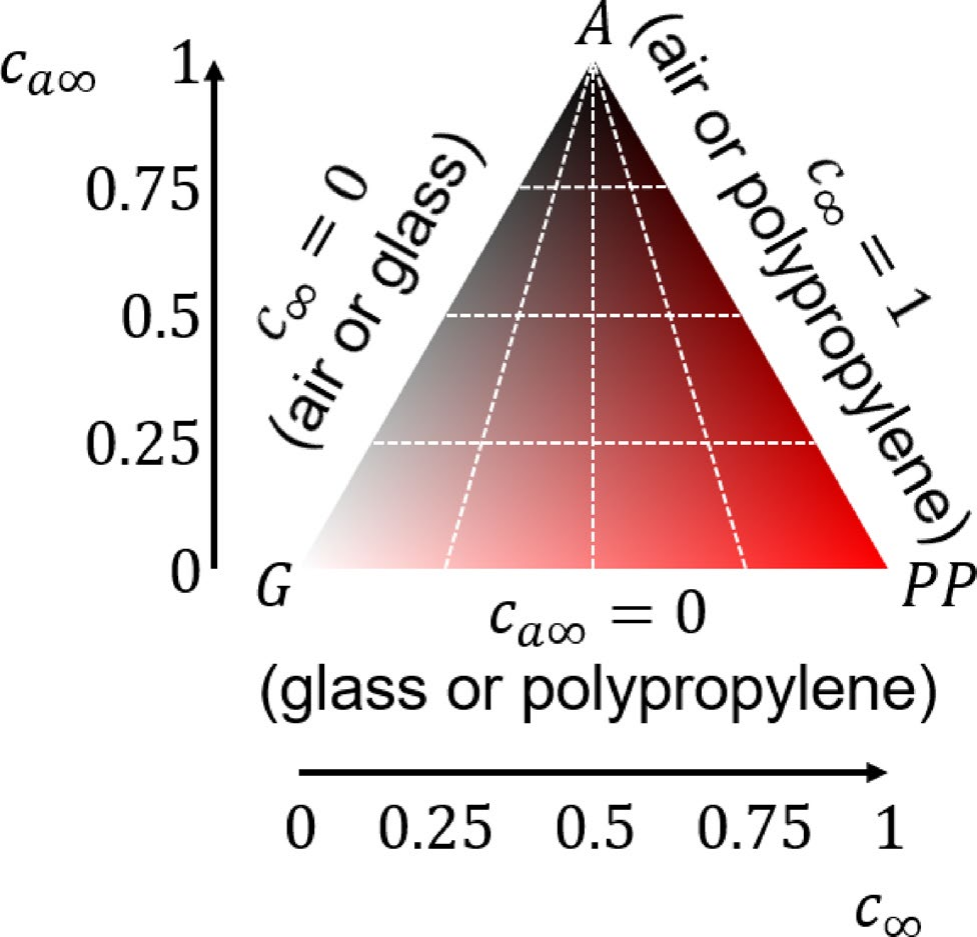
Color-coded phase diagram of all possible steady-state air phase field ($\:{c}_{a\infty\:}$) and steady-state polypropylene concentration field ($\:{c}_{\infty\:}$) values. $\:{c}_{a\infty\:}$ is color-coded by brightness and increases linearly from bottom (bright) to top (black). Along the horizontal lines, $\:{c}_{a\infty\:}$ is constant. $\:{c}_{\infty\:}$ is color-coded by percentage of red and increases linearly from the left (gray) to the right side (red) of the triangle. Along the straight lines connecting vertex *\:A* with the side *\:G-PP*, $\:{c}_{\infty\:}$ is constant. At the three vertices *\:A*, *\:G* and *\:PP* the corresponding phases (air, glass, polypropylene) are pure.

The integral values $\:{\stackrel{-}{c}}_{\infty\:}$ and $\:{M}_{\infty\:}$, are obtained with Eq. (15) for $\:Q=\stackrel{-}{c}$ and *\:Q=M*. Similarly to the instantaneous case, all concentration information is contained in the two fields $\:{c}_{a\infty\:}$ and $\:{c}_{\infty\:}$. Yet, these two fields become interpenetrating in the steady-state as they can take any value between zero and unity. For this reason, we introduce the following phase diagram in Fig. 3 to conveniently bring these two fields together. The air phase field corresponds to the brightness of each pixel, between black ($\:{c}_{a\infty\:}=1$) and brightest ($\:{c}_{a\infty\:}=0$). The steady-state polypropylene concentration field corresponds to the color of each pixel which ranges from white ($\:{c}_{\infty\:}=0$) to red ($\:{c}_{\infty\:}=1$). In Fig. 1a-c, the raw camera images show non‑uniform illumination across the bed. This does not pose a problem, as it is handled during segmentation through manual selection of appropriate color thresholds. We also note that particles at the free surface are visible behind the first layer in contact with the transparent front wall. In the figure, the bed free surface is not yet level. Within the first drum rotation, the bed levels out and the background particles quickly vanish from the images. The algorithms used to transform the camera images into a ternary matrix, including application examples, are described in the authors’ previous works^64,71^.

#### Velocity field

To first estimate an instantaneous velocity field $\:\boldsymbol{u}\left(\boldsymbol{x},t\right)$ from the camera and UFXCT data, we resort to particle image velocimetry (PIV). The method estimates an experimental velocity field by using cross-correlation between two consecutive images. PIV is now an established method for granular flows^100^ including those in a rotating drum^33,101^. PIVlab^102^ is presently used. For the side of the square interrogation area, we choose a length between two and four particle diameters^103^, depending on the investigated scenario. The spatial and temporal resolution of the experimental videos are then used to convert the velocity in pixels per frame to physical units. Further information related to the PIVlab settings can be found in the raw data^104^. A more sophisticated method to extract velocity fields from the experimental data^101^ could have been used. We found that PIVlab delivered realistic and reliable data.

A difficulty with the DEM velocity data lies in turning the discrete particle velocity cloud $\:\left\{{\boldsymbol{X}}_{i}\right(t),{\boldsymbol{V}}_{i}(t\left)\right\}\:$into a continuous Eulerian field $\:\boldsymbol{u}(\boldsymbol{x},t)$, where the index *\:i* ranges from unity to the total number of particles, $\:{n}_{g}+{n}_{p}$. Following the smoothing procedure in^105^, the instantaneous velocity field is calculated as

17 $$\:\boldsymbol{u}(\boldsymbol{x},t)=\frac{1}{W\left(\boldsymbol{x},t\right)}\sum\:_{i=1}^{{n}_{g}+{n}_{p}}{w}_{i}\left(\boldsymbol{x},t\right){\boldsymbol{V}}_{i}\left(t\right),$$

where $\:{w}_{i}$ is a smoothing kernel given by

18 $$\:{w}_{i}\left(\boldsymbol{x},t\right)=\mathrm{exp}\left(-\left[{\left(\frac{x-{X}_{i}\left(t\right)}{{r}_{x}}\right)}^{2}+{\left(\frac{y-{Y}_{i}\left(t\right)}{{r}_{y}}\right)}^{2}+{\left(\frac{z-{Z}_{i}\left(t\right)}{{r}_{z}}\right)}^{2}\right]\right),$$

with $\:{r}_{x}={r}_{y}=0.72{d}_{p}$ and $\:{r}_{z}=0.5{d}_{p}$ being the smoothing radii. Because we are only interested in two-dimensional slices of the bed in the drum direction, $\:{r}_{z}$ is different. The sum of all smoothing kernels in Eq. (17) is given by

19 $$\:W\left(\boldsymbol{x},t\right)=\sum\:_{i=1}^{{n}_{g}+{n}_{p}}{w}_{i}\left(\boldsymbol{x},t\right).$$

Equations (17)-(19) describe a Gaussian smoothing of all particle velocities using all DEM images between $\:{t}_{0}$ and $\:{t}_{1}$. The time-average velocity field, $\:{\boldsymbol{u}}_{{\infty\:}}\left(\boldsymbol{x}\right)$, is defined by substituting $\:Q=\boldsymbol{u}$ in Eq. (15) for both the DEM and experimental data.

## Results and dΙscussions

### Polypropylene concentration and mixing index

**Fig. 4:**
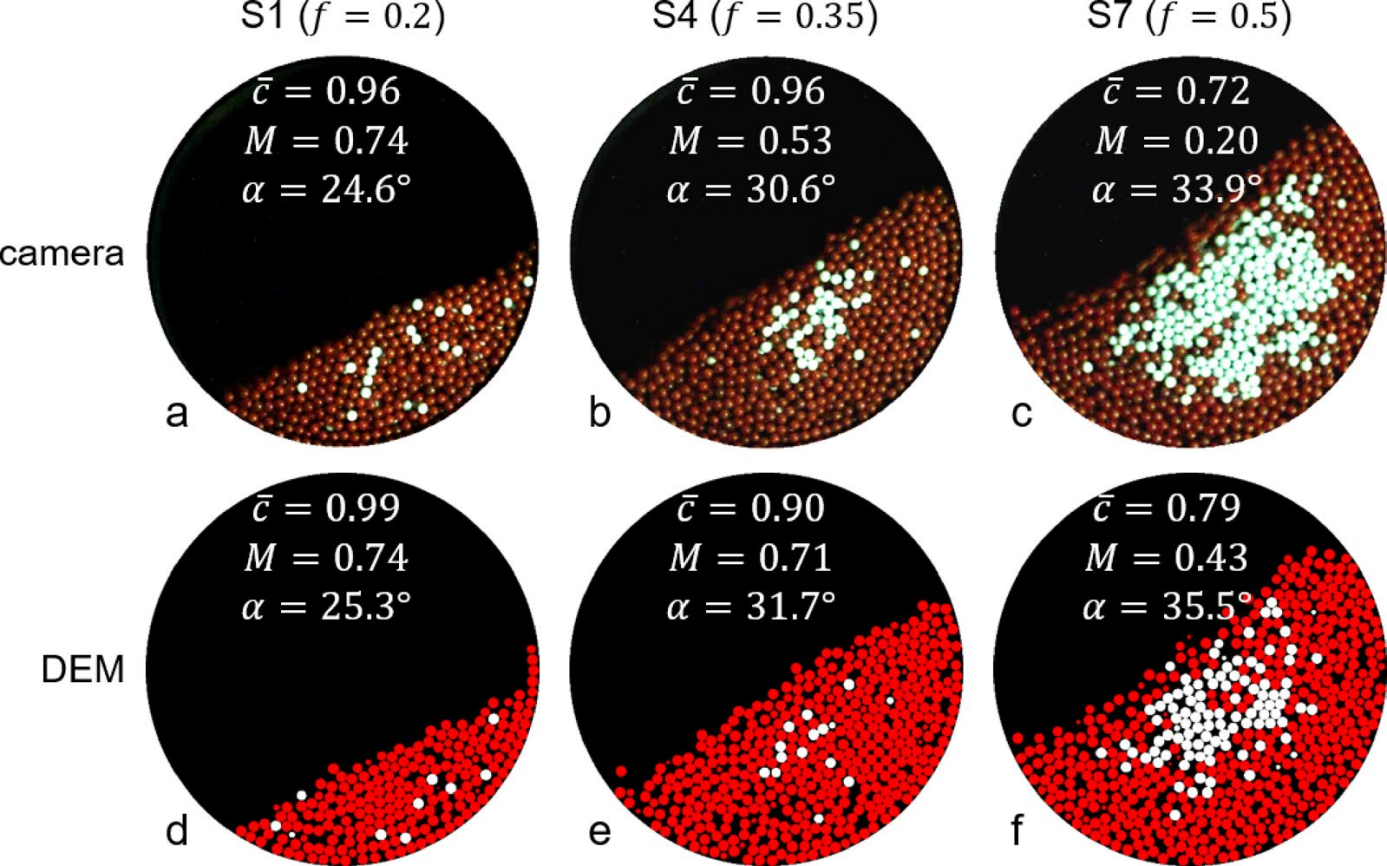
Steady-state images in scenarios S1, S4 and S7 after twenty drum rotations. Camera images **(a-c)** are taken at the front-end and DEM image slices **(d-f)** at drum depth $\:z={d}_{p}/2$.

First, instantaneous camera and UFXCT images taken in the steady-state are qualitatively compared to the DEM results. Figure 4 shows front-end camera images in the top row and the corresponding DEM images in the bottom row. Camera and DEM front-end data are taken at depth *\:z=0* and $\:z={d}_{p}/2$, respectively. In all three scenarios, that are S1, S4 and S7, the white core of glass particles is well visible. The fraction of white particles forming the core, given by the glass concentration $\:1-\stackrel{-}{c}$, tends to increase with the filling degree. In Fig. 4a and c, it equals 0.04 for the lowest filling degree, that is *\:f=0.2*, and 0.28 at the highest filling degree, that is *\:f=0.5*. The instantaneous shape of the bed free surface can also be seen in Fig. 4. The shapes in the DEM images agree with their camera counterparts in all three scenarios. The slight sigmoid shape of the free surface in scenario S7, well visible in Fig. 4c and f, shows that the flow is between the rolling and cascading regimes. The flat shape of the free surface in scenarios S1 and S4 suggest flow in the rolling or the sliding regime.

**Fig. 5:**
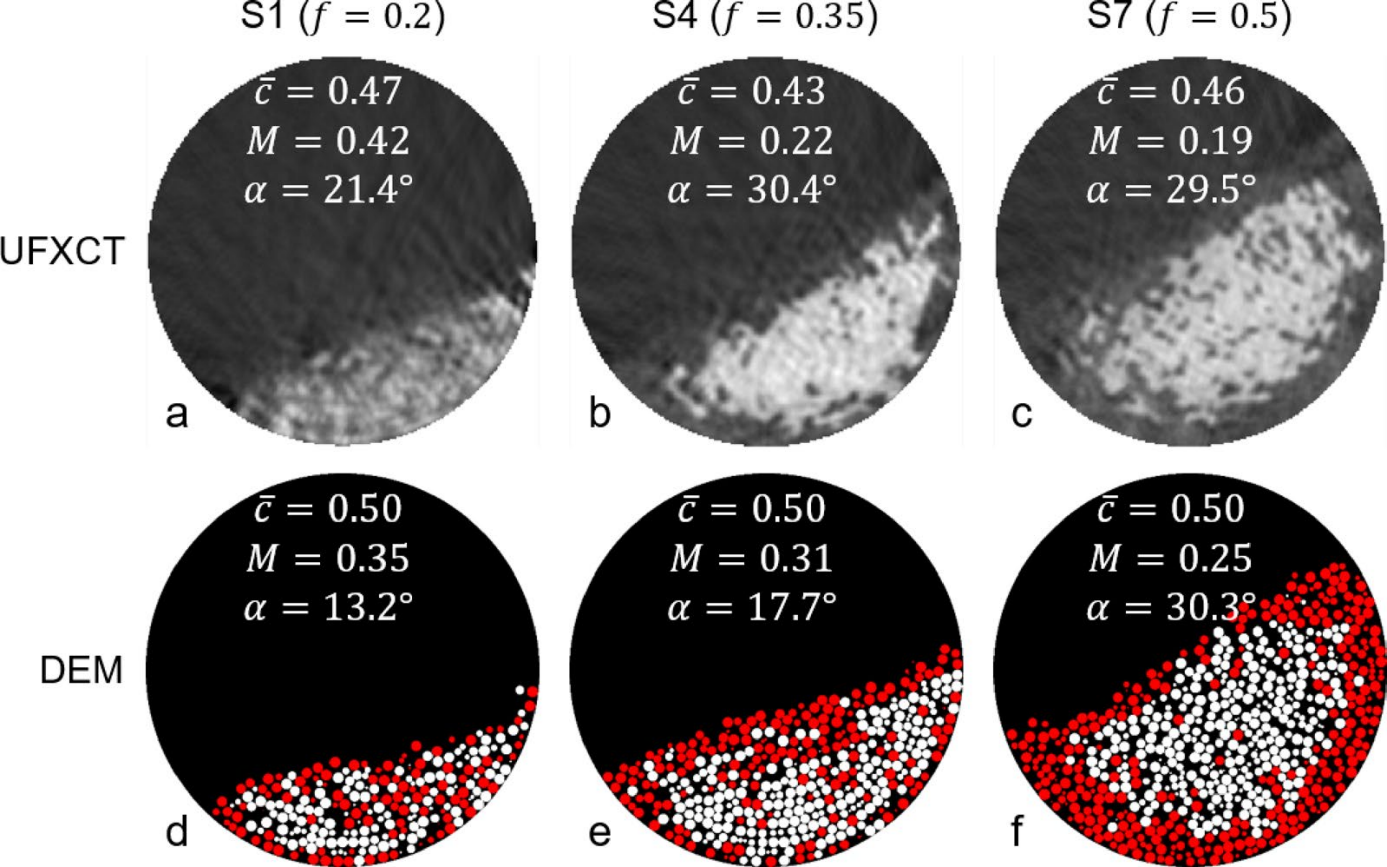
Steady-state images in scenarios S1, S4 and S7 after twenty drum rotations. UFXCT images **(a-c)** and DEM image slices **(d-f)** are obtained at drum depth *\:z/L=0.5*.

In a similar way, the top and the bottom rows in Fig. 5 respectively show slice images in the bulk taken with the UFXCT and DEM, both at depth *\:z/L=0.5*. In the three presented scenarios, it is visually clear from Figs. 4 and 5 that the segregated white core is larger in the bulk than at the front-end. For instance, the white concentration, given by $\:1-\stackrel{-}{c}$, equals 0.21 in Fig. 4f (front-end) and 0.50 in Fig. 5f (bulk). It is also clear from the Figures that the dynamic angle of repose ($\:\alpha\:$) is greater at the front-end than in the bulk. For instance, $\:\alpha\:$ equals 35.5° in Figs. 4f and 30.3° in Fig. 5f. There is a fairly good qualitative agreement between experiments and simulations in terms of filling degree and dynamic angle of repose, both for the front-end and the bulk. The average difference in dynamic angle of repose for all three scenarios is 4% in Figs. 4 and 26% in Fig. 5.

**Fig. 6:**
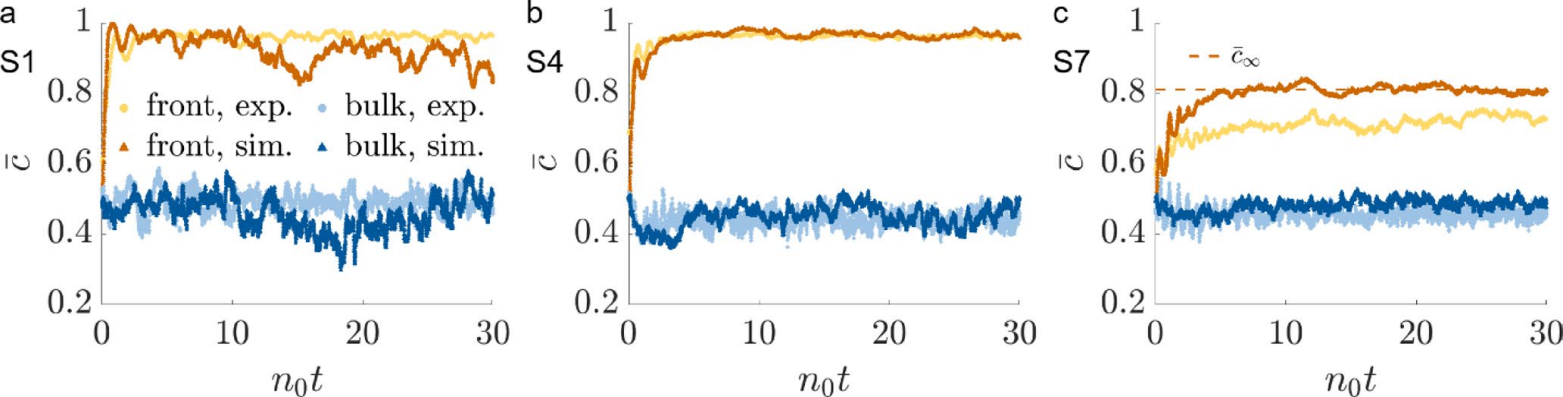
Spatially averaged polypropylene concentration ($\:\stackrel{-}{c}$) at the front-end (*\:z/L=0*) and in the bulk (*\:z/L=0.5*) as a function of drum rotations ($\:{n}_{0}t$) for (a) S1 (*\:f=0.20*), (b) S4 (*\:f=0.35*), (c) S7 (*\:f=0.50*). The dash line in subplot (c) indicates the steady-state spatially averaged concentration ($\:{\stackrel{-}{c}}_{\infty\:}$).

Moving to a quantitative assessment, we now compare the spatially averaged polypropylene concentration $\:\stackrel{-}{c}(z,t)$ and the mixing index *\:M(z,t)* as a function of time for scenarios S1, S4 and S7 at the front-end and in the bulk. The comparative results related to $\:\stackrel{-}{c}$ are shown in Fig. 6. Time (*\:t*) is expressed in terms of number of drum rotations ($\:{n}_{0}t$) by multiplying it with the drum rotational speed ($\:{n}_{0}$). The orange curves show the data at the drum front-end and the blue ones at the mid-section. All DEM curves agree relatively well with their experimental counterparts during the initial transient and steady-state. At the front-end, the polypropylene concentration starts from its initial value, that is 0.5, and rises to a greater steady-state value $\:{\stackrel{-}{c}}_{\infty\:}$ because of end-wall induced axial segregation^46^. In scenarios S1 and S4, the polypropylene concentration reaches a steady-state after about 4 rotations, and in S7 after about 8 to 10 rotations. In the bulk, the concentration remains practically constant over time. The steady-state spatially averaged concentration in the bulk ($\:{\stackrel{-}{c}}_{\infty\:}$) turns out to be slightly smaller than 0.5. In Fig. 6b, for instance, we find $\:{\stackrel{-}{c}}_{\infty\:}=0.45$. This is expected because of particle mass conservation and the higher $\:{\stackrel{-}{c}}_{\infty\:}$ value at the front-end.

The curves $\:\stackrel{-}{c}\left({n}_{0}t\right)$ in Fig. 6 exhibit some fluctuations, both for the simulations and the experiments. The vertical slices extracted from the DEM simulations do not pass through the particle centers. As a result, the spheres appear in each examined *\:z*-plane as disks of varying size. This can be seen in Fig. 5d-f. Axial, out-of-plane particle motion causes these disks to change size and to appear or disappear over time. We attribute the concentration fluctuations in the simulation to this natural diffusive axial particle motion. Two observations support this idea. First, these fluctuations are larger in the bulk (dark blue curves), where axial motion is more pronounced, than at the front (dark orange curves). Second, the fluctuations in the bulk are larger in S4 than in S7. With increasing thickness of the passive layer, a larger fraction of the particles in the slice moves as a rigid body. Hence, collisional axial displacements become less likely in S7. The scenario S1 is excluded, as it is not in the rolling regime. We further note that the magnitude of the fluctuations cannot be compared quantitatively between simulations and experiments. The camera images capture the axial motion only partially. The bed, once in motion, is not visible beyond the front layer and the UFXCT images contain an inherent noise contribution unrelated to particle motion.

**Fig. 7:**
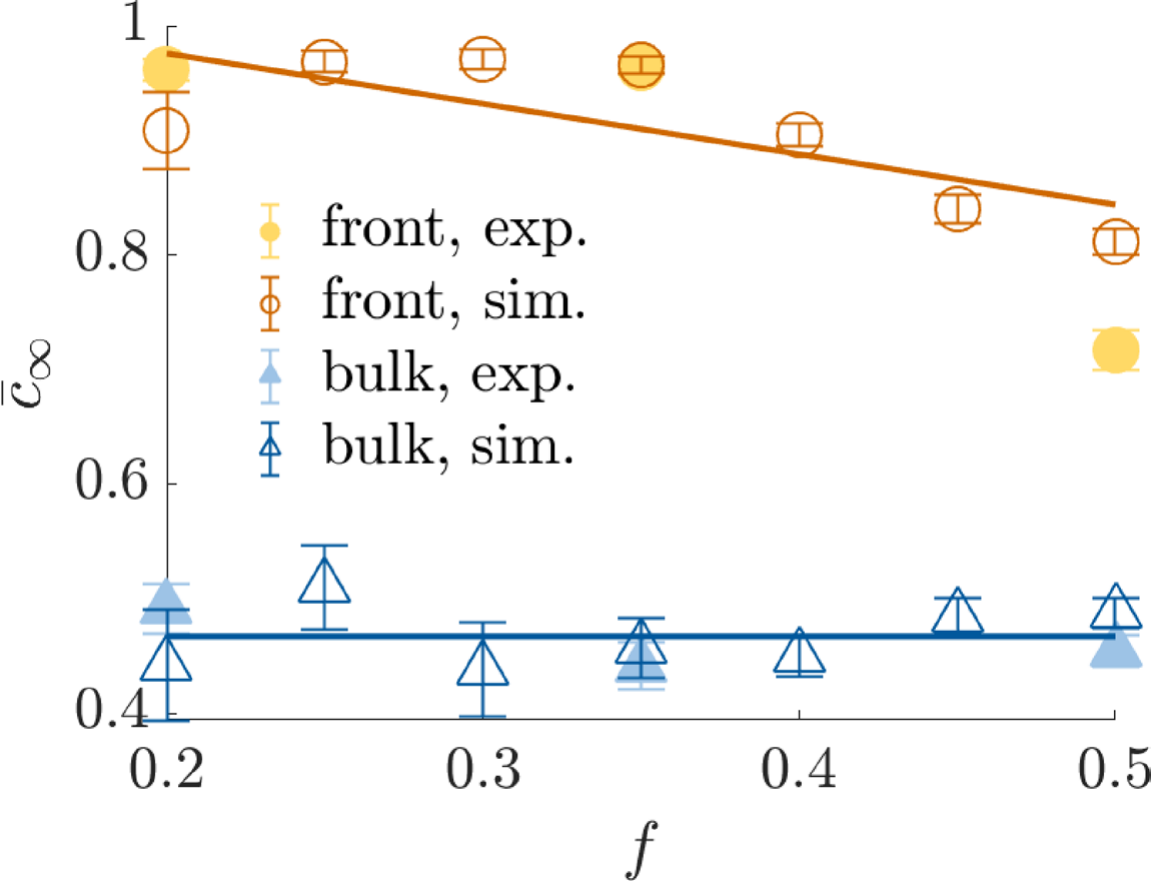
Steady-state spatially averaged polypropylene concentration ($\:{\stackrel{-}{c}}_{\infty\:}$) as a function of the filling degree (*\:f*) at the front-end (*\:z/L=0*, orange) and in the bulk (*\:z/L=0.5*, blue). The unfilled and filled symbols correspond to DEM and experimental data. The error bars indicate the standard deviation of the steady-state values. The straight lines are best-fitted to the DEM data.

The two orange curves in Fig. 6c show that the steady-state spatially averaged concentration $\:{\stackrel{-}{c}}_{\infty\:}$ at the front-end is smaller in scenario S7 than in S1 and S4. This suggests a decreasing migration of heavy glass particles from the front-end towards the bulk with increasing filling degree *\:f*. To examine this in more detail, $\:{\stackrel{-}{c}}_{\infty\:}$ is plotted as a function of the filling degree in Fig. 7. All other scenarios, for which only DEM data exist, are also included. The two straight lines of the form $\:{\stackrel{-}{c}}_{\infty\:}={a}_{c}f+{b}_{c}$ in Fig. 7 are fitted to the DEM data using linear regression. The coefficients along with the correlation coefficients of the linear fit ($\:{R}_{c}^{2}$) are listed in Table 4. At the front-end, the linear fit shown in orange, decreases as the filling degree increases. At low filling degree, that is *\:f<0.4*, individual values of $\:{\stackrel{-}{c}}_{\infty\:}$ nears unity, indicating an almost depletion of white particles from the front-end, as seen in Fig. 4a-b, d-e. The heavier white particles migrate from the front-end towards the bulk and are replaced by light red particles. In scenario S7, that is the highest filling degree, $\:{\stackrel{-}{c}}_{\infty\:}$ decreases down to 0.8. Here, a sizeable core of glass particles is still visible at the front-end (Fig. 4c, f). In the bulk, $\:{\stackrel{-}{c}}_{\infty\:}$ is equal to about 0.47 irrespective of the filling degree. Taking scenarios S1, S4 and S7 into account, the mean average difference in $\:{\stackrel{-}{c}}_{\infty\:}$ between experiment and simulation is 15∙10^− 3^ (2.6%) at the front-end and 0.023∙10^− 3^ (0.3%) in the bulk.

**Table 4:** Parameters of the linear-fit to the steady-state spatially averaged polypropylene concentration ($\:{\stackrel{-}{c}}_{\infty\:}={a}_{c}f+{b}_{c}$) and mixing index ($\:{M}_{\infty\:}={a}_{M}f+{b}_{M}$) as a function of the filling degree (*\:f*). $\:{R}^{2}$ is the correlation coefficient. In the bulk, we fit a horizontal line to the concentration data, so $\:{R}_{c}^{2}$ is zero.

	$\:{a}_{c}$	$\:{b}_{c}$	$\:{R}_{c}^{2}$	$\:{a}_{M}$	$\:{b}_{M}$	$\:{R}_{M}^{2}$
Front	−0.44	1.06	0.54	−1.85	1.38	0.79
Bulk	0	0.47	0	−1.04	0.74	0.93

**Fig. 8:**
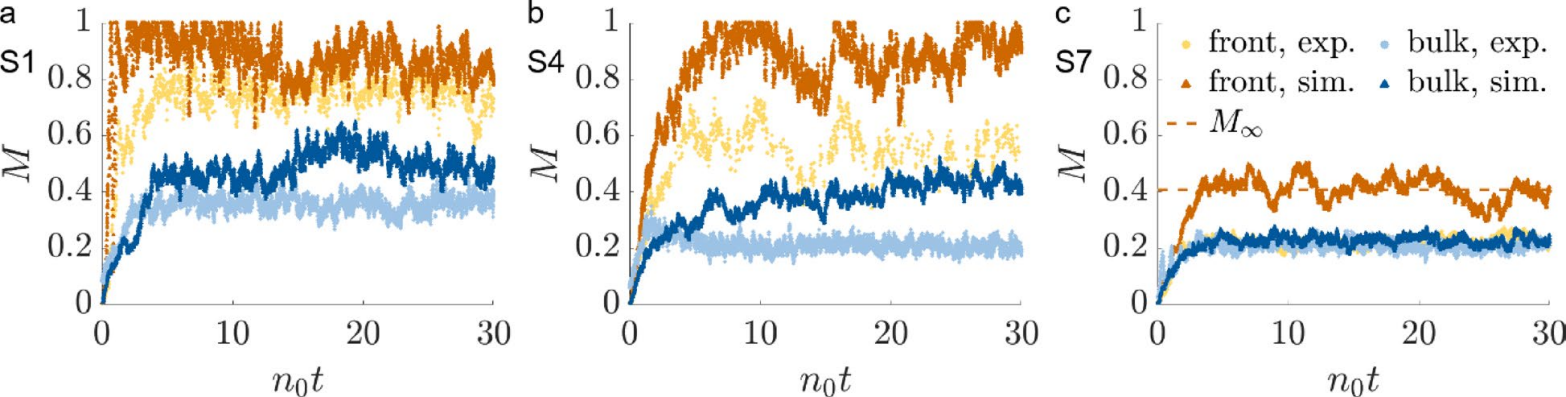
Mixing index (*\:M*) at the front-end (*\:z/L=0*) and in the middle cross-sectional plane in the bulk (*\:z/L=0.5*) as a function of drum rotations ($\:{n}_{0}t$) for (a) S1 (*\:f=0.20*), (b) S4 (*\:f=0.35*), (c) S7 (*\:f=0.50*).

We now examine the evolution of the mixing index for the same three scenarios S1, S4 and S7. Figure 8 shows *\:M* against time. Each curve starts from zero and increases to eventually reach a steady-state, denoted by an average mixing index $\:{M}_{\infty\:}$, around which it fluctuates. All curves reach the steady-state after about ten rotations. In the three scenarios, the mixing index in both the transient and the steady-state is greater at the front-end than in the bulk. This is attributed to the presence of heavier particles in the bulk, which form a more compact segregated core, as seen in Fig. 5. This leads to a smaller contact length between the two granular species. In scenarios S1 and S4, the yellow and orange curves determined at the front-end strongly fluctuate. We quantify the fluctuations with the moving standard deviation. Using an abscissa window size of one rotation, the moving standard deviation at the front-end is on average equal to 0.052 and 0.047 for S1 and S4. The strong fluctuations in S1 and S4 occur because $\:\stackrel{-}{c}$ is close to unity (Fig. 7). This in-turn means that $\:{L}_{\mathrm{m}\mathrm{a}\mathrm{x}}\approx\:{L}_{\mathrm{m}\mathrm{i}\mathrm{n}}$, which makes Eq. (14) sensitive to small changes in the images. As seen in Fig. 4d-e, the front-end mixing in S1 and S4 are limiting cases. On many occasions, there are not enough white particles and so, the denominator $\:{L}_{\mathrm{m}\mathrm{a}\mathrm{x}}-{L}_{\mathrm{m}\mathrm{i}\mathrm{n}}$ in Eq. (14) tends to zero. Numerical errors in Eq. (14) occasionally result in a mixing index greater than unity. These erroneous values are replaced with unity. In scenario S7, where $\:\stackrel{-}{c}$ becomes smaller, these numerical errors disappear and the moving standard deviation at the front-end lessens to 0.017. In the bulk, where $\:\stackrel{-}{c}$ is close to 0.5, the curves fluctuate even less. The moving standard deviation equals on average 0.027, 0.021 and 0.014 for S1, S4 and S7.

**Fig. 9:**
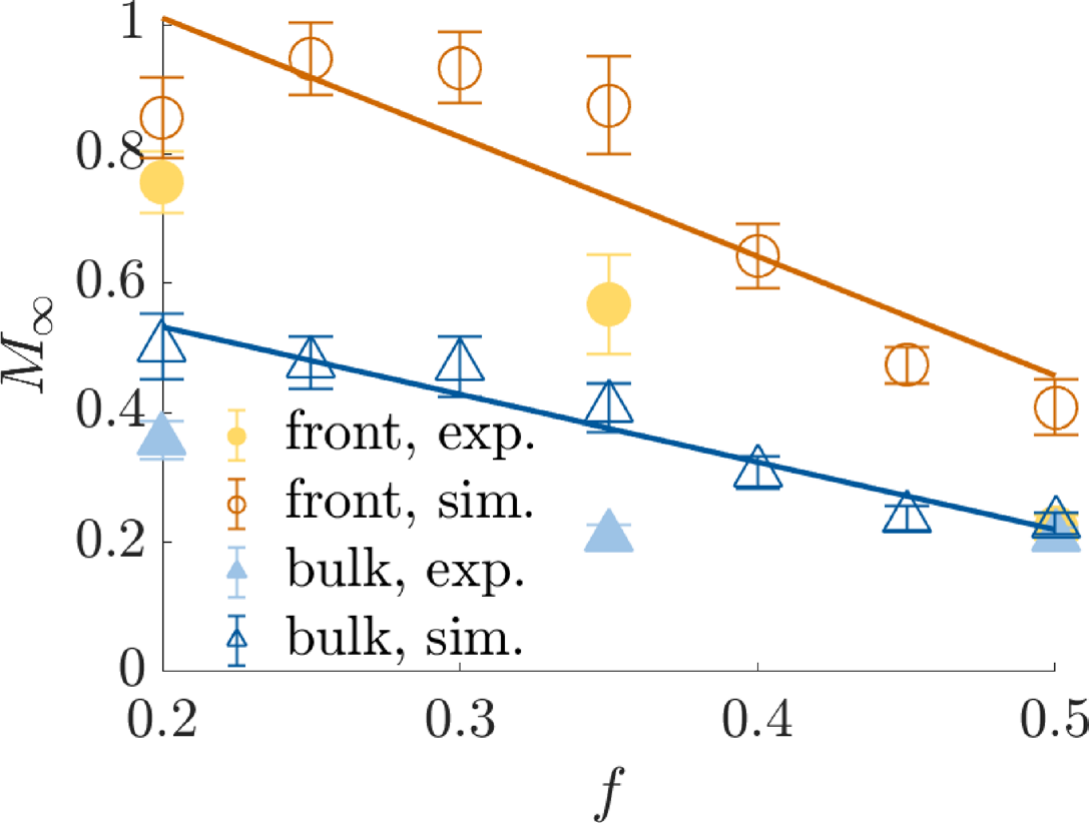
Steady-state mixing index ($\:{M}_{\infty\:}$) as a function of filling degree (*\:f*) at the front-end (*\:z/L=0*) and in the bulk (*\:z/L=0.5*). The unfilled symbols correspond to simulation data and the filled symbols to experimental data. The error bars indicate the standard deviation of the steady-state values. The straight lines are best-fitted to the DEM data.

To further analyze the mixing, the steady-state value $\:{M}_{\infty\:}$ is plotted in Fig. 9 as a function of the filling degree (*\:f*) for all scenarios. Straight lines of the form $\:{M}_{\infty\:}={a}_{M}f+{b}_{M}$ are also fitted to the DEM data using linear regression. At the front-end and in the bulk, $\:{M}_{\infty\:}$ generally decreases with increasing filling degree, similarly to the DEM results of Widhate et al.^106^ for a binary system with size difference. At the front-end, the steady-state mixing index initially maintains a relatively constant value $\:{M}_{\infty\:}\approx\:0.9$ for *\:f<0.35*. Beyond that, $\:{M}_{\infty\:}$ decreases linearly to about 0.41. In the bulk, $\:{M}_{\infty\:}$ decreases much more linearly throughout the entire filling degree range. It starts from about 0.5 and goes down to 0.3. In agreement with Fig. 7, $\:{M}_{\infty\:}$ is smaller in the bulk than at the front-end. The difference in $\:{M}_{\infty\:}$ between the front-end and the bulk gets smaller as the filling degree increases from 0.2 to 0.5. The mean average difference in $\:{M}_{\infty\:}$ between experiment and simulation is greater than in $\:{\stackrel{-}{c}}_{\infty\:}$. Taking scenarios S1, S4 and S7 into account, it is equal to 0.20 (50%) at the front-end and 0.12 (49%) in the bulk.

### Steady-state concentration field

**Fig. 10:**
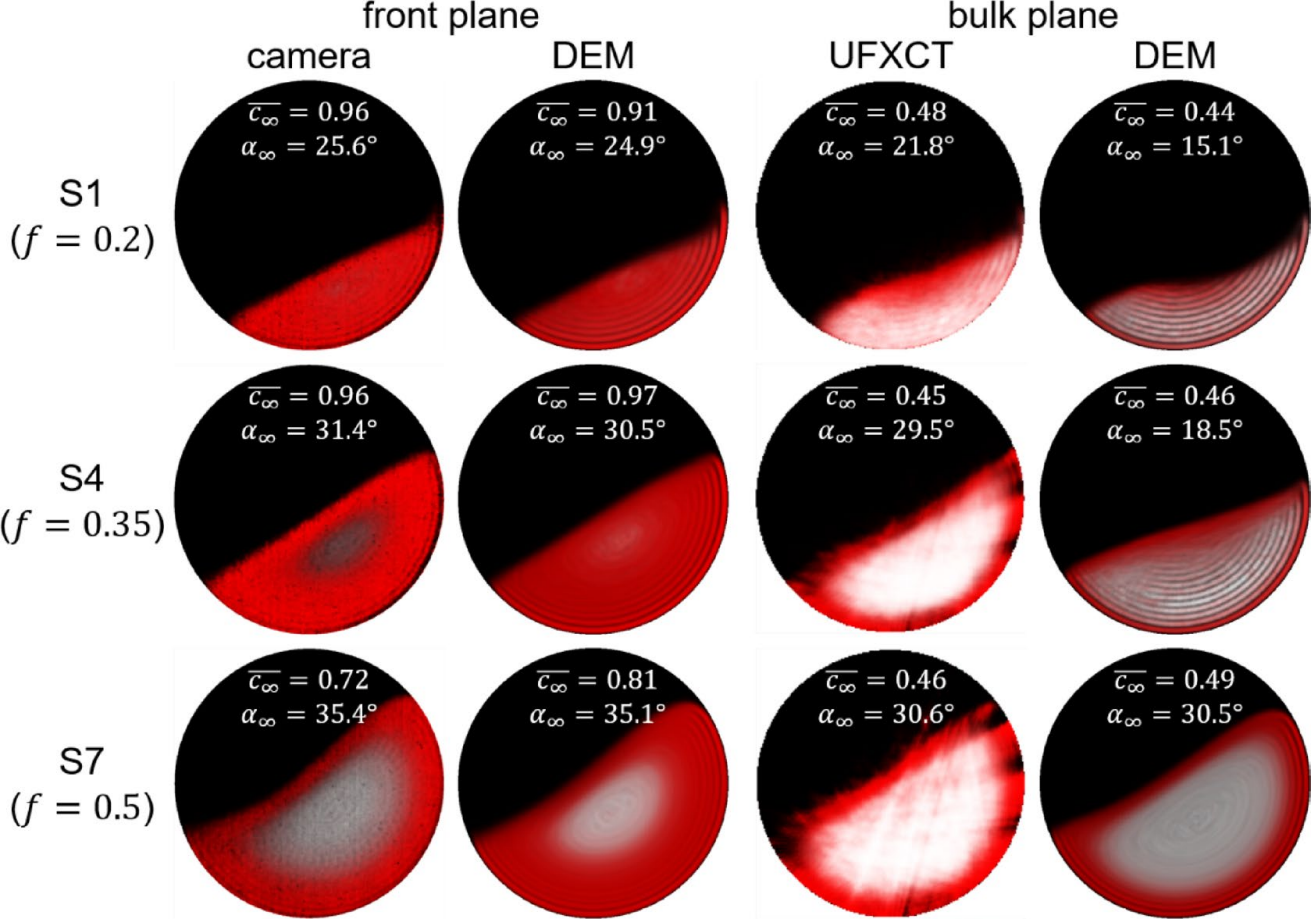
Time-averaged steady-state concentration profiles for the three scenarios (S1, S4, S7) at the front-end (*\:z/L=0*) and in the bulk (*\:z/L=0.5*). The experimental profiles at the front-end are from averaging camera images, at the bulk plane from UFXCT images. For the color coding see Fig. 3.

In this section, we compare the phase fields in the steady-state. We first show the time-averaged air phase field $\:{c}_{a\infty\:}$ and the polypropylene concentration $\:{c}_{\infty\:}$ at the front-end and in the bulk. Figure 10 compares these two fields for scenarios S1, S4 and S7 using the color triangle previously defined in Fig. 3.

**Fig. 11:**
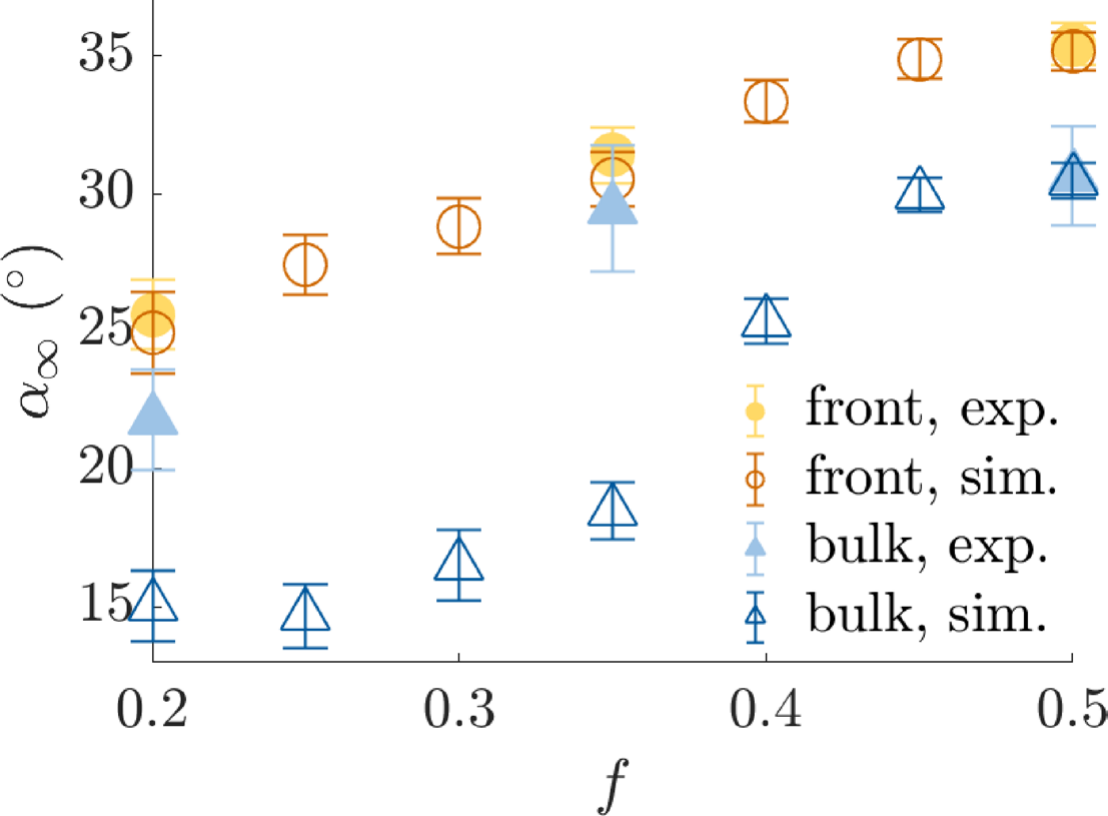
Dynamic angle of repose ($\:{\alpha\:}_{{\infty\:}}$) as a function of filling degree (*\:f*) at the front-end (*\:z/L=0*) and in the bulk (*\:z/L=0.5*). The unfilled symbols correspond to simulation data and the filled symbols to experimental data. The error bars indicate the standard deviation of the steady-state values.

A good agreement between experiment and simulation is here observed. The agreement is better at the front-end than in the bulk. The dynamic angle of repose ($\:{\alpha\:}_{\infty\:}$) in each experimental image agrees well with that from the DEM simulations. The angle difference between experiment and simulation is on average 0.6° (2%) at the front-end and 6.0° (23%) in the bulk. The angle difference is bigger in scenarios S1 (6.7°, 31%) and S4 (11.0°, 37%) in the bulk. Angle differences of similar magnitude are also reported in the literature^55^. For the case of S1 this difference can be partly attributed to UFXCT artefacts, due to which air above the particle bed is instead classified as polypropylene. This can be seen by visually comparing the two images in Fig. 10 (S1 bulk). We also see in Fig. 10 that, irrespective of the scenario, the dynamic angle of repose at the front-end is greater than in the bulk, in line with previous investigations in a unary system^107^. This holds true for all seven DEM scenarios, as seen in Fig. 11, where the dynamic angle of repose is plotted against the filling degree for all scenarios. The angle is greater at the front-end due to the additional friction with the circular cap^107^. Using the average of the experimental and simulation values in Fig. 10, we estimate $\:{\alpha\:}_{\infty\:}=$ 25.3°, 31.0° and 35.3° at the front-end and $\:{\alpha\:}_{\infty\:}=$ 18.4°, 24.0° and 30.6° in the bulk for S1, S4 and S7, respectively. We conclude that the dynamic angle of repose increases with the filling degree in both planes. This trend is also observed if one only considers either the experimental or the DEM data in each plane, as seen in Fig. 11. We attribute this increase in $\:{\alpha\:}_{\infty\:}$ with the filling degree at a given *\:z*-plane to the higher particle-drum wall friction, caused by the higher normal stress exerted when the bed consists of more granules, although other effects, such as local composition and segregation patterns, may also contribute. The shape of the free surface in Fig. 10 also agrees between experimental and DEM data at the front-end. Similar to Fig. 4, in S1 and S4 the free surface is a straight line, characteristic of the sliding or rolling regime, whereas in S7 it turns into slightly sigmoid, transitioning from rolling to cascading. The shape of the free surface also agrees between experimental and DEM data at the bulk plane for scenario S1. The noise and artefacts associated with UFXCT do not permit a similar comparison at the bulk plane for scenarios S4 and S7. Concentric arcs describing the trajectories of particles moving with the drum are also seen in Fig. 10. They are prominent in scenarios S1 (all images) and S4 (DEM images). Similar arcs are also observed in the passive layer in RPT and PEPT experiments and DEM simulations^43,52^.

Regarding segregation, the core of heavy glass particles, surrounded by polypropylene particles, is visible in most images. We quantify the size of the segregated core, similarly to the instantaneous images in Figs. 4 and 5, with $\:1-\stackrel{-}{{c}_{\infty\:}}$. Here $\:\stackrel{-}{{c}_{\infty\:}}\left({z}_{0}\right)$ is the spatial average of the time-averaged polypropylene concentration field $\:{c}_{\infty\:}$, at plane $\:z={z}_{0}$. It is given, in analogy to Eq. (13), by

20 $$\:\stackrel{-}{{c}_{\infty\:}}\left({z}_{0}\right)=\frac{\underset{\mathbb{D}\left({z}_{0}\right)}{\overset{}{\iint\:}}{c}_{p\infty\:}\mathrm{d}x\mathrm{d}y}{\underset{\mathbb{D}\left({z}_{0}\right)}{\overset{}{\iint\:}}\left({c}_{p\infty\:}+{c}_{g\infty\:}\right)\mathrm{d}x\mathrm{d}y}.$$

The concentration $\:\stackrel{-}{{c}_{\infty\:}}\left({z}_{0}\right)$ defined in Eq. (20), only used here, differs from the concentration $\:{\stackrel{-}{c}}_{\infty\:}\left({z}_{0}\right)$ earlier defined in Eq. (15). In Eq. (20), the space averaging is performed after the time averaging, whereas in Eq. (15), the order of averaging is reversed. In reality, the difference between $\:\stackrel{-}{{c}_{\infty\:}}\left({z}_{0}\right)$ and $\:{\stackrel{-}{c}}_{\infty\:}\left({z}_{0}\right)$ defined in Eq. (15) is less than 0.3%, 2.5% and 0.1% for camera measurements, UFXCT measurements and DEM simulations respectively. At the front-end, the core is practically nonexistent in scenario S1 ($\:1-\stackrel{-}{{c}_{\infty\:}}=0.04$) and increases in size as the filling degree increases in scenario S7 ($\:1-\stackrel{-}{{c}_{\infty\:}}=0.28$). In the bulk, the size of the segregated core as a fraction of the bed cross-sectional area is more or less constant in all scenarios ($\:1-\stackrel{-}{{c}_{\infty\:}}=0.54\pm\:0.02$). These observations agree with the trends observed in Fig. 7, showing the steady-state spatially averaged polypropylene concentration ($\:{\stackrel{-}{c}}_{\infty\:}$) against filling degree (*\:f*).

**Fig. 12:**
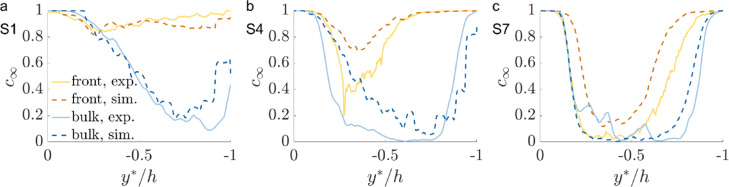
Steady-state polypropylene concentration ($\:{c}_{\infty\:}$) along the line segment *\:AB* (see Fig. 2) at the front-end (*\:z/L=0*) and in the bulk (*\:z/L=0.5*) for scenario (a) S1 (*\:f=0.2*), (b) S4 (*\:f=0.35*), (c) S7 (*\:f=0.5*). $\:{y}^{*}/h=0$ is the free surface and $\:{y}^{*}/h=-1$ is the drum wall.

For a more quantitative comparison, we now look at the polypropylene concentration $\:{c}_{\infty\:}$ along the line segment *\:AB*, previously defined in Fig. 2. The polypropylene concentration is shown in Fig. 12 against the depth of the bed $\:{y}^{*}$, normalized with the maximal steady-state bed height *\:h*. In the three scenarios, S1, S4 and S7, the simulation curves follow the same trend as the ones from the experiments. As one moves from the free surface towards the bottom of the drum, that is from $\:{y}^{*}/h=0$ to $\:{y}^{*}/h=-1$, $\:{c}_{\infty\:}$ starts from unity, decreases towards zero in the white particle core, and increases again towards unity at the wall. The mean difference in $\:{c}_{\infty\:}$ along the segment *\:AB* between simulation and experiment for all three scenarios is on average about 15% both at the front-end and in the bulk. The DEM results in Fig. 12 hence compare relatively well to the experimental data. This provides a good basis for discussing the velocity field $\:{\boldsymbol{u}}_{\infty\:}$.

### Steady-state velocity field

#### Qualitative comparison

**Fig. 13:**
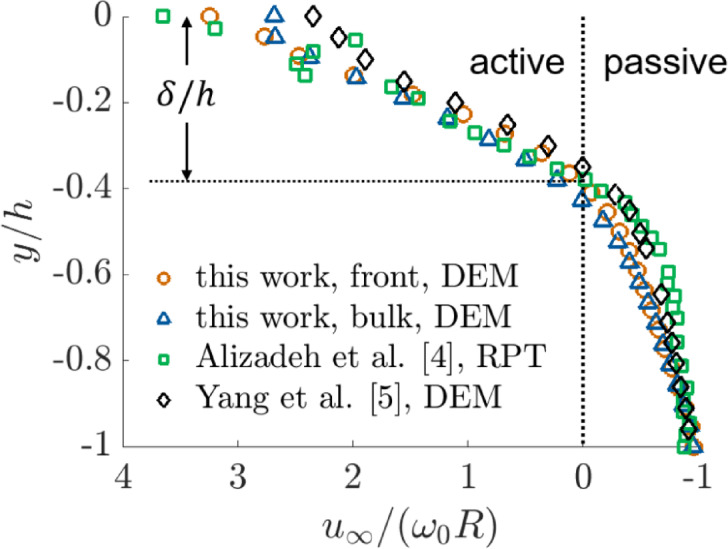
Time-averaged steady-state velocity field ($\:{\boldsymbol{u}}_{\infty\:}$) for the three scenarios (S1, S4, S7) at the front-end (*\:z/L=0*) and in the bulk (*\:z/L=0.5*). Orange vectors indicate the velocity along the line segment *\:AB*. Regions of unreliability and ballistic regions are shaded with grey and blue.

We now shift our attention to the steady-state velocity profiles ($\:{\boldsymbol{u}}_{\infty\:}$) for the three scenarios S1, S4, S7, which are shown at the front-end and in the bulk in Fig. 13. The velocity vectors are shown in blue. We also highlight in orange the steady-state velocity profile along the line segment *\:AB*. To facilitate a qualitative comparison, the blue vectors are scaled so that the one with the highest velocity has the same magnitude in each slice. The orange vectors are scaled in a similar way. The boundary at which the velocity equals zero is well seen in all twelve slices of Fig. 13. In the rolling and cascading regime, this boundary separates the active from the passive layer, which are well-established in the literature^5,108^.

With the grey areas in the experimental slices in Fig. 13, we highlight the regions of unreliability. They mostly concentrate in the top of the particle bed and near the wall. In the top of the bed, especially near the free surface, relatively large particle displacements occur over two consecutive images. In addition, beam hardening artefacts and lower density contrast between polymer and air (as opposed to glass and polymer) contribute to further unreliability in the PIV measurements. Near the bottom wall, there are on average many more red particles, which adversely affects the PIV measurements. The wall vibration also leads to image artefacts near the wall. The blue areas in the DEM slices show the ballistic regions, where particles tend to be free falling for a short time. This seems to contradict the literature at first. The speed in the top of the bed is normally highest near the middle point *\:A*, that is at position $\:{x}^{*}=0$ on the free surface. This is supported by RPT and DEM measurements^4,8^ with a filling degree *\:f=0.5*. In the limiting scenario S1, the bed height is low and so, some particles manage to escape the bed at the apex ($\:{x}^{*}=-{L}^{*}$) and eventually freefall back onto the free surface. In scenario S4, the ballistic motion is weaker and in scenario S7, where the filling degree is highest, the ballistic particle motion no longer happens.

#### Comparisons to literature data (Scenario S7)

**Fig. 14:**
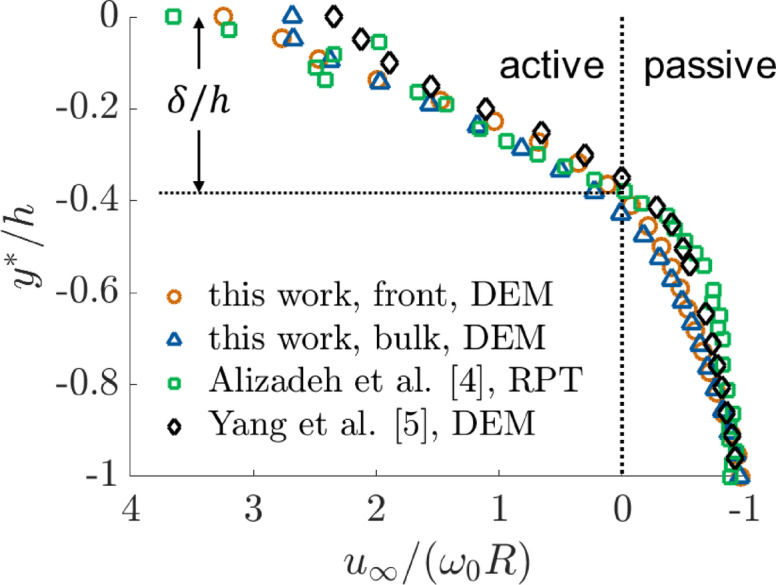
DEM steady-state streamwise velocity along the line segment *\:AB* for scenario S7 (*\:f=0.5*) compared with literature data for the same filling degree. The vertical line indicates the transition from the active ($\:{u}_{\infty\:}>0$) to the passive ($\:{u}_{\infty\:}<0$) layer.

 We first focus on scenario S7, that is the one with the greatest filling degree, because it is one of the most investigated scenarios in the literature. In this way, the orange velocity profile along the segment *\:AB* can easily be compared. For the sake of clarity, we select the front-end and bulk velocity profiles from our DEM simulation and compare them to RPT^4^ and DEM data^5^ obtained from a unary system and a binary system with size difference, both in the rolling regime. The UFXCT and camera data are examined in Sect. “Comparison between experiments and DEM”. As seen in Fig. 14, our DEM results agree with the literature data along the entire segment *\:AB*.

**Fig. 15:**
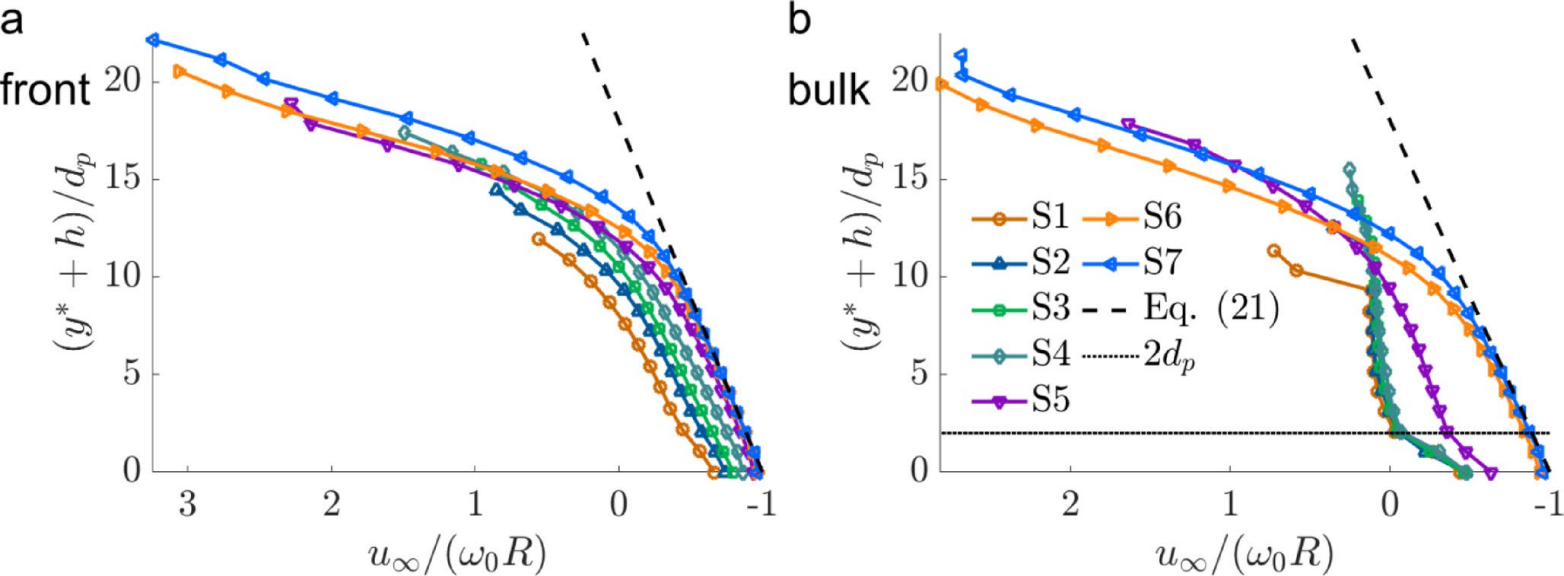
Steady-state streamwise velocity ($\:{u}_{\infty\:}$) along the line segment *\:AB* for all DEM scenarios, **(a)** at the front-end and **(b)** in the bulk. The lines are a guide for the eye.

For the sake of comparison, the ordinate $\:{y}^{*}$ is normalized with the bed height (*\:h*). The abscissa $\:{u}_{\infty\:}$ is normalized with the drum speed at the wall ($\:{\omega\:}_{0}R$). We see, that the increase in $\:{u}_{\infty\:}$ as a function of the depth $\:{y}^{*}$ is linear in both the passive and the active layer, with a different shear rate ($\:\dot{\gamma\:}=\partial\:{u}_{\infty\:}/\partial\:{y}^{*}$) in each layer. In the passive layer, the particles tend to move with the drum as a rigid body^19,29,108^. Ideally, this implies that along the line segment *\:AB* ($\:{x}^{*}=0$),

21 $$\:{u}_{\infty\:}\left({x}^{*}=0,{y}^{*}\right)={\omega\:}_{0}\cdot\:\left({y}^{*}-R+h\right)$$

or $\:{\dot{\gamma\:}}_{p}={\omega\:}_{0}$, where $\:{\dot{\gamma\:}}_{p}$ is the shear rate in the passive layer. The normalized shear rate in the passive layer, $\:{\dot{\gamma\:}}_{p}/{\omega\:}_{0}$, equals here about 1.2, close to the theoretical value of unity and in line with the literature, as seen in Table 5. For the shear rate in the active layer ($\:{\dot{\gamma\:}}_{a}$), the normalized value $\:{\dot{\gamma\:}}_{a}/{\omega\:}_{0}$ equals here 6.5 ± 0.7, which is about 25–50% less than the literature (see Table 5). The shear rates calculated from the camera and UFXCT data for scenario S7 are also shown in Table 5. In both planes, the normalized passive layer shear rate ($\:{\dot{\gamma\:}}_{p}/{\omega\:}_{0}$) is within 20% from unity. The shear rate in the active layer cannot be calculated at the front-end because of data unreliability. In the bulk, the experimental normalized active layer shear rate is about 50% less than its DEM counterpart. Despite that, all the normalized active layer shear rates calculated in both planes are well greater than those in the passive layer, following qualitatively the trend in the literature. In Fig. 14 (S7, bulk), $\:\delta\:$ is the flowing layer thickness associated with the rolling regime. We define it as the depth $\:{y}^{\mathrm{*}}$ at which the streamwise velocity $\:{u}_{{\infty\:}}$ equals zero. Other definitions exist in the literature. In^109,110^, $\:\delta\:$ is the depth at which the measured velocity profile deviates by 1% from the rigid-body profile. Such a definition would also be applicable to scenarios S6 and S7, where the velocity profiles follow Eq. (21) over a substantial portion of the bed depth in Fig. 15. It is nevertheless interesting to compare our data with those by Wang et al.^110^, who obtained $\:\delta\:$ for Froude numbers $\:Fr\approx\:3\cdot\:{10}^{-3}$ and $\:8\cdot\:{10}^{-3}$, and a particle-to-drum-diameter ratio *\:d/D=37.5*. In their work, the authors report layer thicknesses $\:\delta\:/d\approx\:8.6$ and *\:9.2*, respectively. Our Froude number, particle-to-drum diameter ratio, and filling degree for S7 are all comparable to theirs. Using $\:\delta\:/h\approx\:0.4$ (Fig. 14) and Table 5, we find $\:\delta\:/d\approx\:7.6$ for the UFXCT measurements and $\:\delta\:/d\approx\:8.6$ for the DEM, in good agreement with the literature.

**Table 5:** Comparison of normalized shear rate with literature data. At the front-end of the drum, the shear rate in the active layer cannot be determined accurately.

	Method	Drum geometry	Axial depth	Froude number	Bed height	Passive layer shear rate	Active layer shear rate
		*\:L/D*	*\:z/L*	$\:Fr\bullet\:{10}^{2}$	*\:h/R*	$\:{\dot{\gamma\:}}_{p}/{\omega\:}_{0}$	$\:{\dot{\gamma\:}}_{a}/{\omega\:}_{0}$
S7 front	DEM	2.1	0	5.9	1.23	1.17	7.23
S7 front	Camera	2.1	0	5.9	1.18	1.22	-
S7 bulk	DEM	2.1	0.5	5.9	1.19	1.22	5.83
S7 bulk	UFXCT	2.1	0.5	5.9	1.05	0.81	3.23
Alizadeh et al^4^.	RPT	0.15	0–1	1.8	0.69	1.04	12.3
Yang et al^5^.	DEM	3	0.5	1.8	0.79	1.24	8.81

#### DEM velocity profiles

Having validated our DEM results against literature for scenario S7, we now compare all DEM scenarios among one another. Figure 15 shows the DEM velocity profiles along the segment *\:AB* (see Fig. 13). The ordinate is expressed here as $\:{(y}^{*}+h)/{d}_{p}$, which equals 0 at point *\:B* and increases as one moves towards *\:A*. One advantage of this rescaling is that the vertical axis depicts the number of particles between any given point and point *\:B* at the drum wall. The black dashed line represents rigid body rotation and is determined using Eq. (21).

The velocity profile at the front-end (Fig. 15a) is qualitatively similar to that associated with the typical rolling regime profile of Fig. 14 for all filling degrees. The passive and active layers are recognizable in all scenarios, with corresponding shear rates $\:{\dot{\gamma\:}}_{p}\approx\:{\omega\:}_{0}$ (parallel to the dashed line) and $\:{\dot{\gamma\:}}_{a}>{\omega\:}_{0}$. Quantitatively, for the highest filling degree (scenario S7), the velocity in the passive layer agrees very well with Eq. (21). Yet, as the filling degree decreases, the discrepancy with Eq. (21) increases due to a decrease in the magnitude of the granular velocity at the wall, $\:{u}_{\infty\:}\left({y}^{*}=-h\right)$. This is attributed to the smaller granular mass, leading to smaller particle-particle and particle-wall friction in the passive layer. This finding is supported by Beaulieu et al.^6^, who found $\:{u}_{\infty\:}\left({y}^{*}=-h\right)/\left({\omega\:}_{0}R\right)\approx\:-1$ for cubic particles but $\:{u}_{\infty\:}\left({y}^{*}=-h\right)/\left({\omega\:}_{0}R\right)\approx\:-0.6$ for spheres. Parker et al.^41^ also observed some slipping at the wall in their PEPT experiments, with $\:{u}_{\infty\:}\left({y}^{*}=-h\right)/\left({\omega\:}_{0}R\right)\approx\:-0.55$ for a filling degree of about 0.35. In scenario S1, we determine here $\:{u}_{\infty\:}\left({y}^{*}=-h\right)/\left({\omega\:}_{0}R\right)\approx\:-0.66$ at the front-end, which is close to the values by^6,41^. Despite this small discrepancy with Eq. (21), in all scenarios the shear rate deviates from $\:{\omega\:}_{0}$ at about half the total bed height as one moves from point *\:B* upwards.

In the bulk (Fig. 15b) one observes two qualitatively different velocity profiles. The profiles for the highest filling degrees (scenarios S6 and S7) match that associated with the rolling regime of Fig. 14. The profiles of the four lowest filling degrees (scenarios S1-S4, $\:f\le\:0.35$), however, collapse to the same curve, which is qualitatively different. Here, as one moves from point *\:B* upwards, the velocity starts at about $\:{-\omega\:}_{0}R/2$, sharply increases along a distance of about two to three particle diameters, and then settles to a positive value close to zero until the free surface. This profile implies that the bottom layer in contact with the drum, defined with a thickness of approximately two to three particle diameters, forms a boundary region with a shear rate of $\:{\dot{\gamma\:}}_{b}>{\omega\:}_{0}$. This boundary region acts as a wall-slip layer between the drum wall and the rest of the particle bed, which in turn constitutes a sliding region, with a shear rate of $\:{\dot{\gamma\:}}_{s}\approx\:0$. The particles in the sliding region fluctuate around their positions and slide towards the positive $\:{x}^{*}$ direction at a speed much smaller than that of the boundary. The particle motion in these two regions is confirmed from visual inspection of the DEM video data. Only the free surface of the bed for scenarios S1 and S2 is an exception to this particle behavior, as the ballistic region at the apex of the bed is large enough to extend until the segment *\:AB* (Fig. 13). This is also visible in the curves for S1 and S2 in Fig. 15b. The velocity profile and particle behavior of scenarios S1-S4 in the bulk correspond to the sliding flow regime^29^, which is observed when the wall friction coefficient ($\:{\mu\:}_{W}$), the filling degree and the Froude number are all small enough. Mellmann^29^ developed a theoretical model for the transition between the sliding and the rolling regimes for unary systems, based on a moment balance around the rotating axis. For constant friction coefficient and Froude number, as is the case here, Mellmann’s model predicts the existence of a critical filling degree ($\:{f}_{c}$), given by

22 $$\:{f}_{c}=\frac{{\theta\:}_{c}-\mathrm{sin}{\theta\:}_{c}}{2\pi\:},$$

where $\:{\theta\:}_{c}$ is the critical filling angle, which satisfies^29^

23 $$\:{\mu\:}_{W}=\frac{4{\mathrm{sin}}^{3}\left(\frac{{\theta\:}_{c}}{2}\right)\mathrm{sin}\left({\alpha\:}_{\infty\:}\right)}{3\left({\theta\:}_{c}-\mathrm{sin}{\theta\:}_{c}\right)\left(1+Fr\right)}.$$

Accordingly, the flow regime is sliding if $\:f<{f}_{c}$ and rolling if $\:f>{f}_{c}$. This is in agreement with Fig. 15b. In fact, the velocity profile of scenario S5 shows the transition between the two flow regimes. The velocity at the wall lies between the two extremes, $\:{-\omega\:}_{0}R/2$ and $\:{-\omega\:}_{0}R$, and a boundary region about two particle layers deep is observed. The rest of the bed can clearly be divided into a passive and an active region with respective shear rates $\:{\dot{\gamma\:}}_{p}\approx\:{\omega\:}_{0}$ and $\:{\dot{\gamma\:}}_{a}>{\omega\:}_{0}$.

According to the same model by Mellmann^29^, $\:{f}_{c}$ is an increasing function of the dynamic angle of repose ($\:{\alpha\:}_{\infty\:}$). This implies the existence of a critical dynamic angle of repose $\:{\alpha\:}_{c}$ such that the regime is sliding if $\:{\alpha\:}_{\infty\:}<{\alpha\:}_{c}$ and rolling if $\:{\alpha\:}_{\infty\:}>{\alpha\:}_{c}$. Comparing the velocity profiles in Fig. 15 with the values of $\:{\alpha\:}_{\infty\:}$ from the DEM simulations in Fig. 11, a value of about $\:{\alpha\:}_{c}\approx\:25^\circ\:$ seems plausible for the given friction coefficient and Froude number of the DEM simulations. The dynamic angle of repose ($\:{\alpha\:}_{\infty\:}$) for a given filling degree is greater at the front-end than in the bulk and increases with filling degree in both *\:z*-planes, as explained already (see Sect. 3.2). This explains why the case $\:{\alpha\:}_{\infty\:}<{\alpha\:}_{c}$, and therefore the sliding regime, is only observed for low filling degrees in the bulk.

#### Comparison between experiments and DEM

**Fig. 16:**
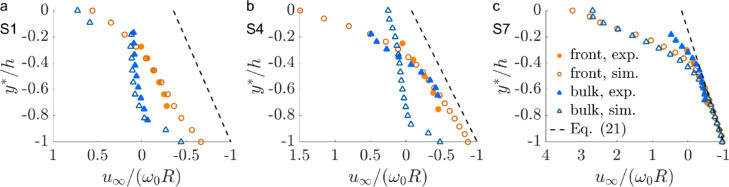
Steady-state streamwise velocity ($\:{u}_{\infty\:}$) along the line segment *\:AB* for scenario (a) S1 (*\:f=0.2*), (b) S4 (*\:f=0.35*), (c) S7 (*\:f=0.5*).

We now move on to comparing the steady-state velocities determined from all methods along the line segment *\:AB*. We consider the scenarios S1, S4 and S7. Only the reliable experimental data (see Sect. 3.3.1) are included in the following analysis. Figure 16 shows the velocity $\:{u}_{\infty\:}$ against the depth $\:{y}^{*}$. The orange data points relate to the front-end and the blue points to the bulk. The black dashed line is determined using Eq. (21).

The experimental velocities match their DEM counterparts in both planes in scenario S1 and at the front-end in S4. In scenario S7, both experimental profiles are rolling regime ones, like the DEM profiles. However, the experimental shear rates in the active region ($\:{\dot{\gamma\:}}_{a}$) are smaller than the ones in the simulations. In general, the DEM velocity profiles at the front-end wall agree well with the camera data, further validating the simulations. The largest disagreement between experiment and DEM is in the bulk data in scenario S4. In this case, the experiment indicates a rolling flow regime, as opposed to the sliding one in the simulation. This could be improved by using more accurate restitution, friction and rolling friction coefficients obtained by calibration^93^. On the other hand, the UFXCT velocity profile in scenario S4 closely matches the DEM and camera profiles of the same scenario at the front-end. A comparison with Fig. 11 shows that the dynamic angles of repose ($\:{\alpha\:}_{\infty\:}$) in these three cases are almost identical, and significantly greater than the DEM $\:{\alpha\:}_{\infty\:}$ in the bulk. This suggests that the experimental data also support the conclusion drawn from the DEM data, that a smaller dynamic angle of repose is associated with the sliding regime and a larger one with the rolling regime.

To further support this claim, we turn our attention to the turning point depth ($\:\delta\:$), defined as the depth at which the streamwise velocity ($\:{u}_{\infty\:}$) changes direction (see Fig. 14), or

24 $$\:{u}_{\infty\:}\left({y}^{*}=-\delta\:\right)=0.$$

We first argue that the turning point depth normalized by the bed height ($\:\delta\:/h$) is an indicator of the flow regime, and then we show that the values of $\:\delta\:/h$ differ significantly depending on the relation between the dynamic angle of repose ($\:{\alpha\:}_{\infty\:}$) and the critical angle ($\:{\alpha\:}_{c}$). In the rolling regime, $\:\delta\:$ is the depth of the active region, which is commonly assumed to be thin in the literature^29,108^. For instance, Alizadeh et al.^4^ measured $\:\delta\:/h$ in the range 24–33% for a half-filled drum in their RPT experiments, and Ding et al.^42^ reported $\:\delta\:/h\approx\:40\:\%$ from their PEPT measurements. Based on that, we have for the rolling regime that

25 $$\:24\%\le\:\frac{\delta\:}{h}\le\:40\%.$$

On the other hand, in the sliding regime $\:\delta\:$ is the thickness of the sliding region and $\:h-\delta\:$ that of the boundary. Since the boundary has a constant thickness of 2 to 3 particles irrespective of the filling degree (see Fig. 15b), one has

26 $$\:h-\delta\:={\lambda\:}_{b}{d}_{p},$$

where $\:{\lambda\:}_{b}$ is the dimensionless thickness of the boundary. Here we use $\:{\lambda\:}_{b}=3$. Starting from Eq. (26), we develop a simple model for the ratio $\:\delta\:/h$ as a function of the filling degree (*\:f*) for the sliding regime as follows. We first define the steady-state filling degree ($\:{f}_{\infty\:}$) similarly to Eq. (1) as

27 $$\:{f}_{\infty\:}=\frac{{\theta\:}_{\infty\:}-\mathrm{sin}{\theta\:}_{\infty\:}}{2\pi\:},$$

where $\:{\theta\:}_{\infty\:}$ is the steady-state filling angle, defined by

28 $$\:{\theta\:}_{\infty\:}=2{\mathrm{cos}}^{-1}(1-h/R).$$

**Fig. 17:**
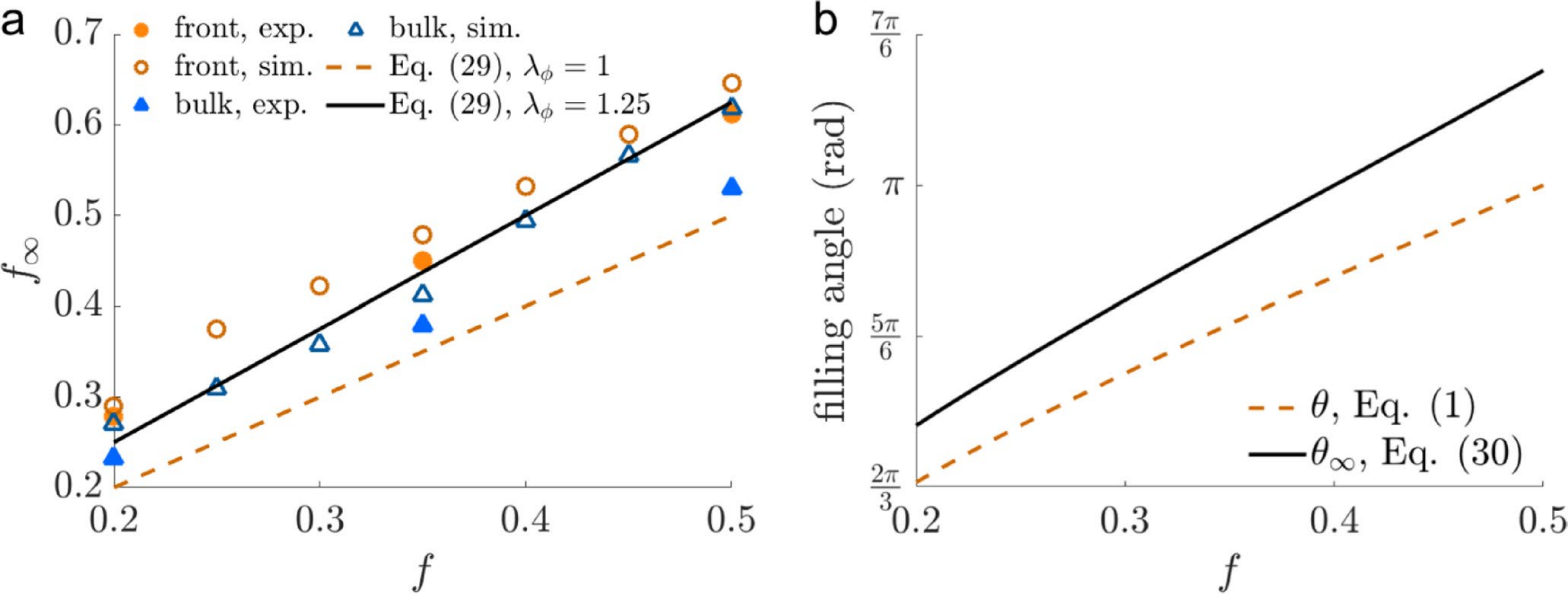
**(a)** Steady-state filling degree ($\:{f}_{{\infty\:}}$) and **(b)** initial ($\:\theta\:$) and steady-state ($\:{\theta\:}_{{\infty\:}}$) filling angle as a function of filling degree (*\:f*). No data points are shown in (b), as the purpose of the plot is to show that $\:{\theta\:}_{{\infty\:}}$ increases with *\:f*.

Due to dilatancy^111,112^, $\:{f}_{\infty\:}$ and $\:{\theta\:}_{\infty\:}$ are expected to be greater than *\:f* and $\:\theta\:$ respectively, which are defined when the bed is at rest. This is verified in Fig. 17a, where the experimental and DEM values of $\:{f}_{\infty\:}$ are plotted as a function of *\:f*. For simplicity we assume that the increase in the bed volume due to dilatancy is proportional to the initial bed volume, or

29 $$\:{f}_{\infty\:}={\lambda\:}_{\phi\:}f,$$

where $\:{\lambda\:}_{\phi\:}>1$ is the packing fraction ratio. From Fig. 17, we see that a value $\:{\lambda\:}_{\phi\:}=1.25$ agrees well with the DEM and camera data. A smaller value closer to unity would agree well with the UFXCT data. Due to the noise of the UFXCT method, the position of the free bed surface is not as reliable as the other data acquisition methods. Therefore, from now on the value $\:{\lambda\:}_{\phi\:}=1.25$ is used. Substituting Eq. (27) into (29), one then has

30 $$\:{\theta\:}_{\infty\:}-\mathrm{sin}{\theta\:}_{\infty\:}=2\pi\:{\lambda\:}_{\phi\:}f.$$

Equation (30) is plotted in Fig. 17b, which shows that $\:{\theta\:}_{\infty\:}$ is an increasing function of *\:f*.

Returning to the sliding regime, we divide Eq. (26) by *\:h* to get

31 $$\:\frac{\delta\:}{h}=1-\frac{{\lambda\:}_{b}{d}_{p}}{h}.$$

Finally, substituting *\:h* from Eq. (28) into the right-hand side of Eq. (31) we get

32 $$\:\frac{\delta\:}{h}=1-\frac{{\lambda\:}_{b}{d}_{p}}{R\left(1-\mathrm{cos}\left(\frac{{\theta\:}_{\infty\:}}{2}\right)\right)}.$$

**Fig. 18:**
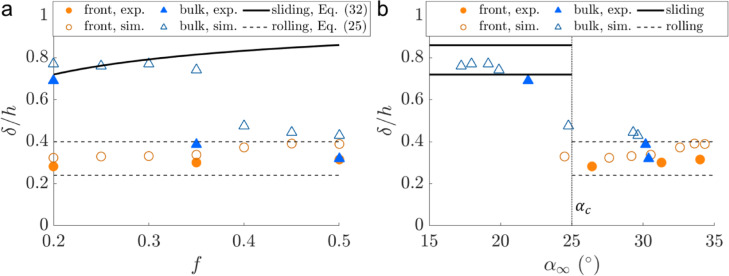
Normalized turning point depth ($\:\delta\:/h$) as a function of **(a)** filling degree (*\:f*) and **(b)** dynamic angle of repose ($\:{\alpha\:}_{\infty\:}$). The vertical line in (b) represents the critical angle, $\:{\alpha\:}_{c}=25^\circ\:$.

The ratio $\:\delta\:/h$ in Eq. (32) is a function of *\:f*, since $\:{\theta\:}_{\infty\:}$ is the unique solution of Eq. (30). Equation (32) gives $\:72\%\le\:\delta\:/h\le\:86\%$ for the sliding regime for *\:f* between 0.2 and 0.5. This is far from the literature range of $\:\delta\:/h$ for the rolling regime, as shown in Fig. 18a. This verifies that the ratio $\:\delta\:/h$ is an indicator of flow regime. The experimental and DEM values of $\:\delta\:/h$ for all scenarios are also plotted in the same figure. The values are all within or close to the range of their respective flow regime, as this was qualitatively assessed from Figs. 15 and 16. The same values of $\:\delta\:/h$ are plotted against the dynamic angle of repose ($\:{\alpha\:}_{\infty\:}$) in Fig. 18b. For angles $\:{\alpha\:}_{\infty\:}$ greater or less than the critical angle $\:{\alpha\:}_{c}\approx\:25^\circ\:$, the value of $\:\delta\:/h$ is in the range of the rolling and the sliding regime, respectively. This is true for all three data acquisition methods. There are two DEM data points for which $\:{\alpha\:}_{\infty\:}$ is very close to $\:25^\circ\:$. The one is from the DEM scenario S5 in the bulk, where the regime is intermediate. The other is DEM scenario S1 at the front plane, whose velocity profile (see Fig. 15a) also approaches the intermediate regime.

## Conclusions

We have studied with UFXCT the granular flow in a horizontal rotating drum and with it, the associated segregation dynamics driven by density difference. The first objective of this work was to assess the applicability of UFXCT for the study of such a physical system. Two different techniques, namely camera and DEM, were used for comparisons. We found that UFXCT captures the transient state of the system, that is the evolution of the polypropylene concentration and the mixing index. UFXCT also captures the steady-state characteristics, both quantitative, such as concentration and velocity fields, and qualitative, such as concentric arcs at low filling, the segregation core and the sliding-rolling regime transition. UFXCT comes with a few challenges. These include the inherent beam hardening effect and the difficulty to accurately distinguish between air and polypropylene within the particle bed because of the relatively small difference in density between these two materials. This introduces uncertainty to the position and shape of the free surface and ultimately makes the velocity field calculated near the free surface rather unreliable. Despite these few shortcomings, UFXCT is a robust experimental technique and is particularly suited to the study of bulk granular flows, which are opaque.

UFXCT experimental data are compared directly with bulk data for the first time in this study. These other bulk data come from DEM simulations, as DEM is an established method. The DEM coefficients were not measured or calibrated. Instead, typical values from the literature were used and the simulation results were validated against front-end wall camera videos. The DEM simulations capture well the salient characteristics of camera measurements, such as polypropylene concentration dynamics and steady-state dynamic angle of repose, concentration and velocity profiles and flow regime. The agreement with the mixing index, where numerical noise occurs, is not as good. However, our DEM results are also in excellent agreement with literature data from a commonly investigated scenario, namely a half-filled drum in the rolling regime. They are also consistent with the literature as far as the relation between the front and bulk planes is concerned. For instance, the bulk plane has a more prominent segregation core^21^ and a smaller dynamic angle of repose^107^ than the front plane. All of the above show that the DEM data are sufficiently reliable for the purpose of comparison with UFXCT. A DEM calibration could certainly improve the general agreement with the experimental data, although it would not alter the main conclusions of our study. In Fig. 4, for example, the number of white glass particles at the front-end is consistently higher than in the corresponding DEM slices. Brandao et al.^27^ also used a DEM to simulate the mixing of two granular species differing in density in a rotating drum. In their calibration, the restitution and friction coefficients were varied over typical literature ranges. They reported a dynamic angle of repose varying from 30° to 42°. This shows that DEM, without a dedicated calibration, remains qualitative rather than quantitative. The mismatch between DEM and experiment also applies to the flow-regime transition (Fig. 16b, bulk scenario S4) and can be attributed to the lack of calibration. In Mellmann’s model for unary systems, see Eq. (23), the critical angle marking the transition between the sliding and rolling regimes depends on the particle-wall friction coefficient. In our system, there are several possible particle-particle and particle-wall pairs, so the transition angle likely depends on all the associated friction coefficients.

The second objective of this work was to investigate the effect of the filling degree on the velocity field and flow regime in a binary system with differing particle densities, thereby demonstrating an application of the UFXCT. We found that, as the filling decreases, the flow regime transitions from rolling to sliding. This transition manifests itself in a change in the dynamic angle of repose. Whether this change is abrupt or gradual could not be resolved in this study. Clarifying it would require additional, costly UFXCT experiments including simulations near the critical point, together with a dedicated DEM calibration. The filling degree associated with this transition differs between the front-end and the bulk of the system due to additional friction at the front cap. This regime transition is consistent with previous experiments performed with unary systems, even though the original assumption of a constant particle density field no longer holds.

## Data Availability

All raw data supporting the present findings of this study are available on the Rossendorf Data Repository [104].

## Latin symbols

*\:c*: instantaneous polypropylene concentration field (-)
$\:\stackrel{-}{c}$: instantaneous spatially averaged polypropylene concentration (-)
$\:{c}_{\infty\:}$: steady-state polypropylene concentration field (-)
$\:{\stackrel{-}{c}}_{\infty\:}$: steady-state spatially averaged polypropylene concentration (-)
$\:\stackrel{-}{{c}_{\infty\:}}$: spatial average of steady-state polypropylene concentration field (-)
$\:{c}_{a}$: instantaneous air phase field (-)
$\:{c}_{a\infty\:}$: steady-state air phase field (-)
$\:{c}_{g}$: instantaneous glass phase field (-)
$\:{c}_{g\infty\:}$: steady-state glass phase field (-)
$\:{c}_{p}$: instantaneous polypropylene phase field (-)
$\:{c}_{p\infty\:}$: steady-state polypropylene phase field (-)
$\:\mathbb{D}$: drum cross-section
*\:D*: drum inner diameter (m)
*\:d*: particle diameter (m)
$\:{d}_{g}$: glass particle diameter (m)
$\:{d}_{p}$: polypropylene particle diameter (m)
$\:{E}^{\mathrm{*}}$: equivalent Young’s modulus (Pa)
$\:{E}_{g}$: glass particle Young’s modulus (Pa)
$\:{E}_{i}$: Young’s modulus of particle *\:i* (Pa)
$\:{E}_{p}$: polypropylene particle Young’s modulus (Pa)
$\:{E}_{w}$: drum wall Young’s modulus (Pa)
$\:{e}_{ij}$: restitution coefficient between particles *\:i* and *\:j* (-)
$\:{F}_{ij}$: contact force exerted by particle *\:j* on particle *\:i* (N)
$\:{F}_{ij}^{n}$: normal contact force exerted by particle *\:j* on particle *\:i* (N)
$\:{F}_{ij}^{t}$: tangential contact force exerted by particle *\:j* on particle *\:i* (N)
*\:Fr*: Froude number (-)
*\:f*: filling degree (-)
$\:{f}_{\infty\:}$: steady-state filling degree (-)
$\:{f}_{c}$: critical filling degree (-)
$\:{f}_{p}$: bed packing fraction (-)
$\:{G}^{\mathrm{*}}$: equivalent shear modulus (Pa)
*\:g*: gravity vector (m/s^2^)
*\:g*: gravity magnitude (9.81 m/s^2^)
*\:h*: bed height (m)
$\:{h}_{0}$: initial bed height (m)
$\:{I}_{i}$: moment of inertia of particle *\:i* (kg∙m^2^)
$\:{k}_{n}$: normal elastic constant (N/m)
$$\:{k}_{r}$$: rolling stiffness (N∙m)
$\:{k}_{t}$: tangential elastic constant (N/m)
*\:L*: drum length (m)
$\:{L}^{*}$: free surface half-length
$\:{L}_{c}$: total length of red/white contact lines (m)
$\:{L}_{\mathrm{m}\mathrm{a}\mathrm{x}}$: maximum contact length (m)
$\:{L}_{\mathrm{m}\mathrm{i}\mathrm{n}}$: minimum contact length (m)
*\:M*: instantaneous mixing index (-)
$\:{M}_{\infty\:}$: steady-state mixing index (-)
$\:{M}_{ij}^{r}$: rolling friction torque exerted by particle *\:j* on particle *\:i* (N∙m)
$\:{m}^{\mathrm{*}}$: equivalent particle mass (kg)
$\:{m}_{g}$: mass of glass particles (kg)
$\:{m}_{i}$: mass of particle *\:i* (kg)
$\:{m}_{p}$: mass of polypropylene particles (kg)
$\:{N}_{g}$: number of glass (white) pixels (-)
$\:{N}_{i}$: number of particles in contact with particle *\:i* (-)
$\:{N}_{p}$: number of polypropylene (red) pixels (-)
$\:{N}_{S}$: number of steady-state images (-)
$\:{n}_{0}$: drum rotational speed (rps)
$\:{n}_{g}$: number of glass particles (-)
$\:{n}_{p}$: number of polypropylene particles (-)
*\:R*: drum inner radius (m)
$\:{R}^{\mathrm{*}}$: equivalent particle radius (m)
$\:{R}_{i}$: radius of particle *\:i* (m)
$\:{R}_{ij}$: vector pointing from the center of particle *\:i* to the point of contact with particle *\:j* (m)
$\:{r}_{x}$: smoothing radius in *\:x*-direction (m)
$\:{r}_{y}$: smoothing radius in *\:y*-direction (m)
$\:{r}_{z}$: smoothing radius in *\:z*-direction (m)
*\:t*: time (s)
$\:{t}_{0}$: start time of steady-state (s)
$\:{t}_{1}$: end time of steady-state (s)
*\:u*: instantaneous velocity field (m/s)
$\:{u}_{{\infty\:}}$: steady-state velocity field (m/s)
$\:{u}_{{\infty\:}}$: steady-state streamwise velocity (m/s)
$\:{V}_{i}$: translational velocity of particle *\:i* (m/s)
$\:{v}_{g}$: glass volume fraction in bed (-)
$\:{v}_{ij}^{n}$: normal relative velocity between particles *\:i* and *\:j* (m/s)
$\:{v}_{ij}^{t}$: tangential relative velocity between particles *\:i* and *\:j* (m/s)
$\:{v}_{p}$: polypropylene volume fraction in bed (-)
$\:{w}_{i}$: smoothing kernel for particle *\:i* (-)
$\:{X}_{i}$: position of particle *\:i* (m)
*\:x*: space coordinate, inertial reference frame (m)
*\:x*: horizontal transverse length (m)
$\:{x}^{*}$: space coordinate, drum reference frame (m)
$\:{x}^{*}$: length in the direction of active layer streamwise velocity (m)
*\:y*: height parallel to gravity (m)
$\:{y}^{*}$: height perpendicular to free surface (m)
$$\:z,\:{z}^{*}$$: axial depth (m)

## Greek symbols

$\:\alpha\:$: instantaneous dynamic angle of repose (rad)
$\:{\alpha\:}_{\infty\:}$: steady-state dynamic angle of repose (rad)
$\:{\alpha\:}_{c}$: critical dynamic angle of repose (rad)
$\:{\alpha\:}_{s}$: static angle of repose (rad)
$\:\beta\:$: DEM parameter related to restitution coefficient (-)
$$\:\dot{\gamma\:}$$: shear rate (s^− 1^)
$\:{\dot{\gamma\:}}_{a}$: active layer shear rate (s^− 1^)
$\:{\dot{\gamma\:}}_{b}$: boundary layer shear rate (s^− 1^)
$\:{\gamma\:}_{n}$: normal viscoelastic damping constant (kg/s)
$\:{\dot{\gamma\:}}_{p}$: passive layer shear rate (s^− 1^)
$\:{\dot{\gamma\:}}_{s}$: sliding region shear rate (s^− 1^)
$\:{\gamma\:}_{t}$: tangential viscoelastic damping constant (kg/s)
$\:{\Delta\:}t$: simulation time step (s)
$\:\delta\:$: active layer depth (m)
$\:{\delta\:}^{{\prime\:}}$: lower boundary of active layer (m)
$\:{\delta\:}_{ij}^{n}$: normal overlap between particles *\:i* and *\:j* (m)
$\:{\delta\:}_{ij}^{t}$: tangential overlap between particles *\:i* and *\:j* (m)
$\:\delta\:{m}_{g}$: glass mass error (kg)
$\:\delta\:{m}_{p}$: polypropylene mass error (kg)
$\:\theta\:$: filling angle (rad)
$\:{\theta\:}_{\infty\:}$: steady-state filling angle (rad)
$\:{\theta\:}_{c}$: critical filling angle (rad)
$\:{\lambda\:}_{b}$: dimensionless boundary thickness (-)
$\:{\lambda\:}_{\phi\:}$: packing fraction ratio (-)
$\:{\mu\:}_{ij}$: friction coefficient between particles *\:i* and *\:j* (-)
$\:{\mu\:}_{ij}^{r}$: rolling friction coefficient between particles *\:i* and *\:j* (-)
$\:{\mu\:}_{W}$: wall friction coefficient (-)
$\:{\nu\:}_{g}$: glass particle Poisson’s ratio (-)
$\:{\nu\:}_{i}$: Poisson’s ratio of particle *\:i* (-)
$\:{\nu\:}_{p}$: polypropylene particle Poisson’s ratio (-)
$\:{\nu\:}_{w}$: drum wall Poisson’s ratio (-)
$\:{\:\rho\:}_{g}$: glass particle density (kg/m^3^)
$\:{\rho\:}_{p}$: polypropylene particle density (kg/m^3^)
$\:{\varOmega\:}_{i}$: angular velocity of particle *\:i* (rad/s)
$\:{\omega\:}_{0}$: drum angular velocity (rad/s)
